# Hypoxia-driven TNS4 fosters HNSCC tumorigenesis by stabilizing integrin α5β1 complex and triggering FAK-mediated Akt and TGFβ signaling pathways

**DOI:** 10.7150/ijbs.86317

**Published:** 2024-01-01

**Authors:** Xinyuan Zhao, Zizhao Mai, Liu Liu, Ye Lu, Li Cui, Jinhua Yu

**Affiliations:** 1 Jiangsu Key Laboratory of Oral Diseases, Affiliated Hospital of Stomatology, Nanjing Medical University, Nanjing, 210029, China.; 2 Stomatological Hospital, School of Stomatology, Southern Medical University, Guangzhou, 510280, China.

**Keywords:** tensin 4, head and neck squamous cell carcinoma, integrin α5β1, HIF-1α, TGF-β

## Abstract

Head and neck squamous cell carcinoma (HNSCC) remains a formidable clinical challenge due to its high recurrence rate and limited targeted therapeutic options. This study aims to elucidate the role of tensin 4 (TNS4) in the pathogenesis of HNSCC across clinical, cellular, and animal levels. We found a significant upregulation of TNS4 expression in HNSCC tissues compared to normal controls. Elevated levels of TNS4 were associated with adverse clinical outcomes, including diminished overall survival. Functional assays revealed that TNS4 knockdown attenuated, and its overexpression augmented, the oncogenic capabilities of HNSCC cells both *in vitro* and *in vivo*. Mechanistic studies revealed that TNS4 overexpression promotes the interaction between integrin α5 and integrin β1, thereby activating focal adhesion kinase (FAK). This TNS4-mediated FAK activation simultaneously enhanced the PI3K/Akt signaling pathway and facilitated the interaction between TGFβRI and TGFβRII, leading to the activation of the TGFβ signaling pathway. Both of these activated pathways contributed to HNSCC tumorigenesis. Additionally, we found that hypoxia-inducible factor 1α (HIF-1α) transcriptionally regulated TNS4 expression. In conclusion, our findings provide the basis for innovative TNS4-targeted therapeutic strategies, which could potentially improve prognosis and survival rates for patients with HNSCC.

## Introduction

Head and neck squamous cell carcinoma (HNSCC) represents a malignancy that originates from the mucosal epithelium within the oral cavity, pharynx, and larynx. Ranking as the sixth most prevalent cancer globally, HNSCC accounts for approximately 700,000 new cases diagnosed each year 1. Numerous risk factors contribute to the initiation and progression of HNSCC, encompassing genetic predisposition, tobacco smoking, alcohol consumption, areca nut chewing, and human papillomavirus infection 2, 3. Despite the availability of diverse treatment modalities, including advanced surgical procedures, radiochemotherapy, targeted therapies, and immunotherapy, the five-year survival rate for HNSCC patients remains disappointingly low 3, 4. This highlights the urgent need to improve our understanding of the molecular mechanisms involved in HNSCC and to identify new therapeutic targets. The development of strategies to tackle these challenges could enhance treatment effectiveness and significantly improve patient outcomes in the management of HNSCC.

The tensin (TNS) gene family comprises four members: TNS1, TNS2, TNS3, and TNS4. The proteins encoded by these genes are localized at the focal adhesions of the plasma membrane, serving as crucial components that bridge the extracellular matrix, actin cytoskeleton, and signal transduction 5, 6. While TNS1, TNS2, and TNS3 exhibit distinct central regions, their domain structures remain relatively similar 7, 8. Conversely, TNS4, also known as COOH-terminus tensin-like protein, is a distinctive member with the smallest molecular mass and lacks the N-terminal common region found in other family members 8. TNS4 has been implicated in numerous vital biological processes, including proliferation, migration, cell adhesion, and apoptosis 9-11. Recent studies indicate that aberrant TNS4 expression is involved in various malignancies, such as breast carcinoma, lung cancer, colorectal cancer (CRC), and gastric cancer 12-16. For example, a long-term clinical trial revealed a significant correlation between elevated TNS4 expression and poor prognosis in breast carcinoma 16. Furthermore, TNS4 interacts with E3 ubiquitin ligase, leading to a reduction in ligand-induced degradation of the epidermal growth factor receptor (EGFR) by decreasing its ubiquitination 17. This mechanism promotes tumor progression and therapeutic resistance by prolonging EGFR signaling. Nevertheless, the specific role of TNS4 in HNSCC warrants further investigation.

In this study, we observe a pronounced increase in TNS4 expression across HNSCC tissues, corroborated by both our in-house cohort and independent public datasets. Elevated TNS4 levels are associated with adverse clinical outcomes in HNSCC patients. Our data reveal that TNS4 depletion diminishes the oncogenic attributes of HNSCC cells in both *in vitro* and *in vivo* models, whereas its overexpression enhances these properties. Mechanistically, TNS4 depletion disrupts the integrin α5β1 complex by inhibiting the interaction between integrin α5 and β1, which results in the attenuation of FAK activity. This, in turn, downregulates the FAK-mediated Akt and TGFβ signaling pathways, suppressing HNSCC tumorigenesis. Additionally, we demonstrate that TNS4 is transcriptionally regulated by hypoxia-inducible factor 1α (HIF-1α). Collectively, these findings underscore the pivotal role of TNS4 in HNSCC pathogenesis and its promising potential as a therapeutic target.

## Materials and methods

### Clinical samples

The study protocol, involving the reuse of clinical samples and related medical information, was approved by the Ethics Committee of the Stomatological Hospital at Nanjing Medical University. All procedures adhered to the Declaration of Helsinki, and written informed consent was obtained from all participants. Detailed clinicopathological features of the in-house HNSCC cohort were summarized in Table S1.

### Cell culture

HNSCC cell lines, UM-SCC-1 (SCC-1), UT-SCC-23 (SCC-23), UMSCC-5, and UMSCC-6, were sourced from the University of Michigan. UM-1 and UM-2 cell lines were procured from the University of California, Los Angeles, while normal human epidermal keratinocytes (NHEKs) were obtained from the ATCC. These HNSCC cell lines were cultured in Dulbecco's Modified Eagle's Medium (DMEM) supplemented with 10% fetal bovine serum, 100 U/mL penicillin, and 100 μg/mL streptomycin. Normal human oral keratinocytes (NHOKs) and NHEKs were maintained in EpiLife media supplemented with human keratinocyte growth supplement (Invitrogen, Carlsbad, CA, USA). All cell lines were cultivated under standard conditions at 37°C in a humidified incubator with 5% CO_2_. Hypoxic conditions were induced using an InvivO_2_ 300 hypoxia workstation (Baker Ruskinn, Ltd., Bridgend, UK) flushed with nitrogen to establish an atmospheric composition of 94% N_2_, 5% CO_2_, and 1% O_2_ at 37°C.

### Lentivirus construction and infection

Oligonucleotides targeting TNS4 (shTNS4 #1, shTNS4 #2) were incorporated into the LV3-pGLV-h1-GFP-puro vector, obtained from GenePharma (Shanghai, China). Simultaneously, the full-length human TNS4 gene was cloned into the pGCL-GFP vector (Genechem, Shanghai, China) to create the TNS4-overexpression construct. Lentiviruses were produced by co-transfecting HEK-293T cells with the recombinant lentiviral vectors, packaging plasmids, and envelope plasmids. After 72 h of incubation, lentiviruses were collected and concentrated. Cancer cells were subsequently transduced with the filtered lentiviruses at a multiplicity of infection of 30. The shFAK was purchased from Santa Cruz Biotechnology (Santa Cruz, CA, USA). The shRNA sequences used are provided in Table S2.

### MTT assay

Cells were seeded in 96-well plates at a density of 3,000 cells/well. At predetermined time points, cells were treated with MTT solution (5 mg/mL, Sigma-Aldrich, Burlington, MA, USA) for 4 h at 37°C. The supernatant was then carefully removed, and the formed formazan crystals were dissolved in 200 μL of dimethyl sulfoxide. The absorbance of the dissolved formazan was measured at 570 nm using the Synergy HT microplate reader (BioTek Instruments, Winooski, VT, USA).

### Colony formation assay

A total of 3,000 cells were seeded per well in 6-well plates. After a 2-week incubation period, cells were fixed using 4% paraformaldehyde, and colonies were visualized by staining with 0.5% crystal violet.

### Sphere formation assay

Cells subjected to specified treatments were seeded in ultra-low adhesion plates (Corning, NY, USA). Briefly, cells were cultured in DMEM/F12 supplemented with 1% B27 supplement (Invitrogen, Carlsbad, CA, USA), 1% N2 supplement (Invitrogen), 20 ng/mL human recombinant epidermal growth factor, and 10 ng/mL human recombinant basic fibroblast growth factor. Tumor spheres formed at the designated time point were imaged using an inverted microscope (Leica Microsystems, Wetzlar, Germany).

### EdU assay

For EdU labeling, cells were incubated in a 10 μmol/L solution for 2 h at 37°C, then fixed with 4% paraformaldehyde and permeabilized with 0.5% Triton X-100. Following this, the cells underwent incubation in a freshly prepared Click-It® Plus reaction cocktail (Invitrogen) for 30 min in the dark at room temperature. After a PBS wash, Hoechst 33342 solution was used to visualize cell nuclei. Images were subsequently captured using an inverted fluorescence microscope (Leica Microsystems).

### Matrigel invasion assay

A matrigel invasion assay was performed using Transwell chambers with an 8 μm pore size (Costar, Cambridge, MA, USA). The upper chambers were seeded with 5.0 × 105 cells/well in serum-free medium, with complete culture medium added to the lower chambers. After a 24 h incubation period, non-invaded cells on the upper side of the membrane were carefully removed with a cotton swab. The invaded cells were fixed with 4% paraformaldehyde and stained with 0.5% crystal violet. Four random fields per chamber were captured, and the average invasion per field was calculated using Image J (https://imagej.nih.gov/ij/index.html).

### Co-immunoprecipitation assay

Following the indicated treatments, cells were lysed in radioimmunoprecipitation assay buffer and centrifuged at 14,000 × g for 20 min. The supernatants were subsequently incubated with primary antibodies and IgG control antibody at 4°C, with gentle agitation overnight. Immunocomplexes were then incubated with magnetic beads (EpiZyme, Shanghai, China) at 4°C, with gentle rocking for 8 h. After washing with elution buffer three times and resuspending in SDS sample buffer, the antigen-antibody-bead complexes were heated to 100°C for 10 min to denature. The samples were subsequently subjected to western blotting.

### Luciferase reporter assay

The indicated TNS4 promoter reporter vector was co-transfected into HNSCC cells along with pcDNA-HIF-1α or pcDNA using the Lipofectamine 3000 (Invitrogen) transfection reagent according to the manufacturer's instructions. Following a 24 h period post-transfection, the relative luciferase activities were assessed with a Dual-Luciferase Assay System (Promega, Madison, WI, USA).

### ChIP-qPCR

The ChIP assays were performed using the ChIP assay kit (Millipore, Billerica, MA, USA). Briefly, cells were treated with 1% formaldehyde for 10 min to cross-link proteins and DNA, after which glycine was added to stop the reaction. After washing twice with PBS, cells were lysed in RIPA buffer and DNA fragments were generated via sonication. For immunoprecipitation, 30 μL Protein A Agarose/Salmon Sperm DNA (50% Slurry) was used to pre-clear the extracted protein. After removing the agarose, cross-linked chromatin fragmentation was subjected to immunoprecipitation using primary antibodies targeting HIF-1α and IgG. After washing with low-salt buffer, high-salt buffer, LiCl wash buffer, and TE buffer, the immunocomplexes were collected and eluted using ChIP elution buffer for 10 min at 65°C. Following incubation with 200 mM NaCl for 4 h at 65°C to reverse cross-linking, the DNA fragments were purified using a DNA Clean/Concentrator kit (Zymo Research, Irvine, CA, USA). Finally, the DNA pellets were resuspended in ultra-pure water and subjected to qPCR.

### Immunohistochemistry (IHC)

Formalin-fixed paraffin-embedded specimens were deparaffinized using xylene and rehydrated through a graded ethanol series. After blocking with goat serum, the sections were incubated with primary antibodies overnight at 4°C. Sections were then washed with PBS three times and incubated with a horseradish peroxidase-conjugated secondary antibody for 1 h at room temperature. Staining signals were detected using a DAB kit. IHC results were quantitatively scored under a microscope by multiplying staining intensity and the percentage of stained cells. The H-score was calculated for each slide using the formula: 3 × percentage of strong staining + 2 × percentage of moderate staining + 1 × percentage of weak staining + 0 × percentage of no staining. Two pathologists blinded to clinical information independently assessed the results.

### Real-time PCR

Total RNA was extracted from cell specimens using the Quick-RNA™ kit (Zymo Research Corp) according to the manufacturer's instructions. SuperScript™ III reverse transcriptase from Invitrogen was used to synthesize cDNA. The cDNA was amplified using Light Cycler 480@SYBR Green I MasterMix (Roche, Applied Sciences, Indianapolis, USA) and detected with a CFX96 Real-Time PCR Detection System (Bio-Rad, Hercules, CA, USA). The 2^-ΔΔCT^ method was employed to analyze relative gene expression changes, using the housekeeping genes GAPDH and β-actin as internal references. The primers used in this study are detailed in Table S2.

### Western blotting

Equal amounts of protein lysates were loaded onto 4%-20% SDS-polyacrylamide gels. Following electrophoresis, separated protein samples were transferred onto 0.2 μm polyvinylidene fluoride membranes using a Trans-Blot Turbo system (Bio-Rad). Membranes were blocked with blocking buffer (EpiZyme, Shanghai, China) at room temperature for 10 min and probed with primary antibodies at 4°C overnight. After washing the PVDF membranes five times for 5 min each with TBST, membranes were incubated with HRP-linked secondary antibodies (Proteintech, Chicago, IL, USA) at room temperature for 1 h. Blot intensities were visualized using Amersham ECL Prime Western Blotting Detection Reagent (Cytiva, Marlborough, MA, USA). The primary antibodies used in this study are as follows: TNS4 (Proteintech), GAPDH (Proteintech), E-cadherin (Proteintech), N-cadherin (Proteintech), PCNA (Proteintech), Vimentin (Proteintech), HIF-1α (Proteintech), Flag (Proteintech), V5 (Proteintech), HA (Proteintech), SNAI2 (Proteintech), FAK (Proteintech), p-Akt (Proteintech), Akt (Proteintech), Smad2 (Proteintech), Smad3 (Proteintech), integrin α5 (Abcam, Cambridge, UK), integrin β1 (Abcam), p-FAK (Cell Signaling Technology, Danvers, MA, USA), p-PI3K (Cell Signaling Technology), PI3K (Cell Signaling Technology), p-Smad2 (Cell Signaling Technology), p-Smad3 (Cell Signaling Technology), TGFβRI (Santa Cruz Biotechnology), TGFβRII (Santa Cruz Biotechnology) and p-Tyr (Santa Cruz Biotechnology).

### Bioinformatic analysis

Public datasets GSE37991, GSE58911, GSE83519, GSE25099, GSE55550, GSE85195, GSE47443, GSE30788, GSE40774, GSE85446, GSE30784, GSE65858, and GSE41613 were obtained from the Gene Expression Omnibus database (https://www.ncbi.nlm.nih.gov/geo/). RNA-seq expression profile data and corresponding clinical information for The Cancer Genome Atlas (TCGA) HNSCC cohort were retrieved from the National Cancer Institute Genome Data Commons (https://gdc.cancer.gov/). The X-tile software (https://medicine.yale.edu/lab/rimm/research/software/) was employed to determine the optimal cutoff point for categorizing HNSCC patients into high and low *TNS4* expression groups. For gene set enrichment analysis (GSEA), HNSCC patients were divided into *TNS4*-high and *TNS4*-low expression groups based on the median value of *TNS4* expression.

### Animal experiments

Animal experiments were performed in compliance with the Institutional Animal Care guidelines and received approval from the Animal Ethics Committee of Southern Medical University. Mice utilized in the study were sourced from the Guangdong Medical Laboratory Animal Center (Foshan, Guangdong, China). In order to establish a 4-nitroquinoline 1-oxide (4-NQO) induced HNSCC murine model, we administered 4-NQO to six-week-old C57BL/6 mice. The 4-NQO was mixed into the drinking water at a concentration of 50 μg/mL and provided to the mice for a period of 16 weeks. Subsequently, the mice were provided with regular drinking water for an additional duration of 8 to 10 weeks. To create a xenograft mouse model, 6-week-old BALB/c nude mice were subcutaneously injected in the dorsal flank with 2 × 10^6^ cells that had undergone the indicated treatments. Throughout the experiment, the tumor volume and health status of the animals were carefully monitored. After four weeks, the mice were euthanized, the tumor xenografts were surgically removed, and the tumors were assessed for size and weight. The excised tumor tissues were fixed and subsequently embedded in paraffin for IHC analysis.

### Statistical analysis

Data analysis for this study was performed using GraphPad Prism 9.0 (GraphPad Software, San Diego, CA, USA). Results are presented as the mean ± standard deviation. Statistical differences between two groups were evaluated using a two-tailed Student's t-test, whereas one-way analysis of variance (ANOVA) was employed for comparisons among multiple groups. Survival curves were plotted using the Kaplan-Meier method, with log-rank tests used for comparative analysis. Pearson correlation analysis was carried out to determine the relationships between different variables. A *P*-value of less than 0.05 was deemed statistically significant.

## Results

### TNS4 upregulation predicts unfavorable outcome in HNSCC

**Figure 1:**
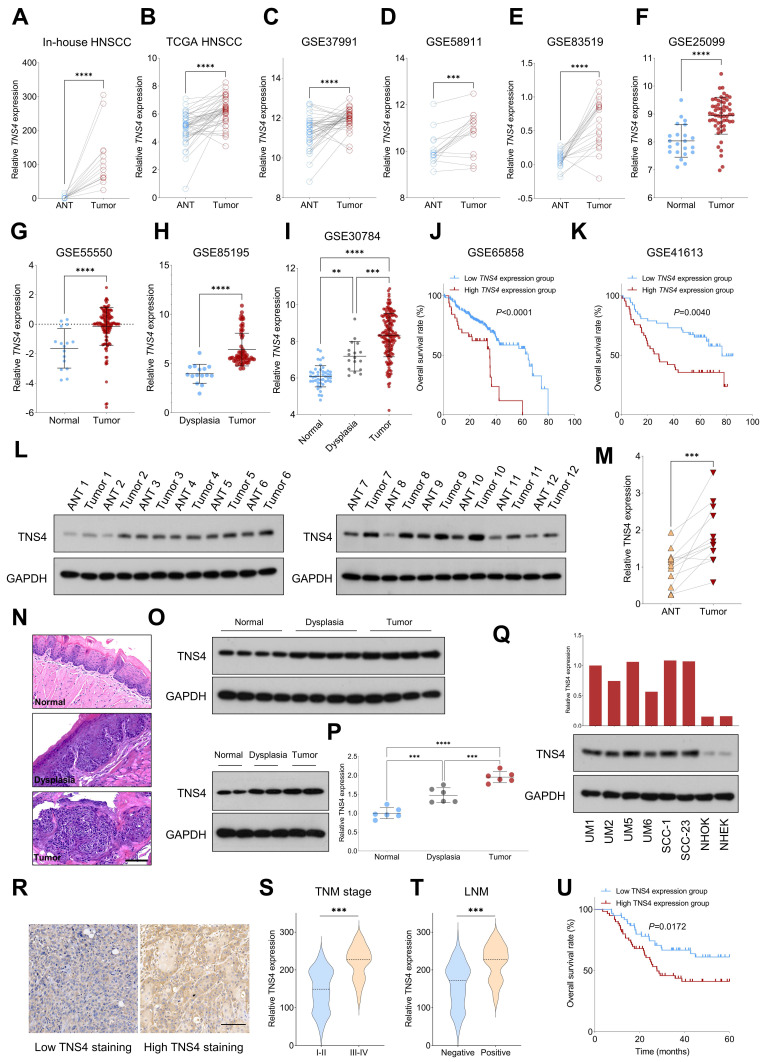
Elevated TNS4 expression is associated with unfavorable prognosis in HNSCC. (A-E) *TNS4* expression in tumor tissues compared to adjacent normal tissues in in-house HNSCC, TCGA HNSCC, GSE37991, GSE58911, and GSE83519. (F-G) The distribution of *TNS4* expression in tumor tissues and normal tissues sourced from GSE25099 and GSE55550. (H) *TNS4* expression in dysplasia tissues versus tumor tissues in GSE85195. (I) *TNS4* expression across normal tissues, dysplasia tissues, and tumor tissues in GSE30784. (J-K) Survival analysis comparing overall survival between HNSCC patients with high and low *TNS4* expression in GSE65858 and in GSE41613. (L-M) Western blot analysis of TNS4 expression in tumor tissues and adjacent normal tissues. (N-P) HE staining and TNS4 expression patterns in normal, dysplastic, and tumor tissues derived from the 4-NQO carcinogenesis model (scale bar=100 μm). (Q) TNS4 protein expression in the indicated HNSCC cell lines, NHOK, and NHEK. (R) Representative images of high and low TNS4 staining in HNSCC tissues (scale bar=100 μm). (S-T) TNS4 staining intensity in tumor tissues of advanced stages (III-IV) compared to early stages (I-II), and in tumor tissues with and without LNM. (U) Survival analysis comparing overall survival between in-house HNSCC patients with high and low TNS4 staining intensity.

We initially assessed the expression pattern of *TNS4* in HNSCC using both in-house and publicly available genome-wide gene expression datasets. Our findings indicated that *TNS4* expression was significantly elevated in tumor tissues compared to adjacent normal tissues (ANTs) across multiple independent HNSCC cohorts, including in-house HNSCC, TCGA HNSCC, GSE37991, GSE58911, and GSE83519 (Figure 1A-1E). We also observed a similar trend in *TNS4* expression in GSE25099 and GSE55550 datasets, where *TNS4* was overexpressed in tumor tissues relative to normal tissues (Figure 1F, 1G). Notably, a marked increase in *TNS4* expression was found in HNSCC tissues compared to dysplastic tissues (Figure 1H). Moreover, *TNS4* expression demonstrated a progressive increase from normal tissues to dysplastic tissues and ultimately to tumor tissues (Figure 1I). Survival analysis of the GSE65858 and GSE41613 datasets revealed that HNSCC patients with high *TNS4* expression levels exhibited significantly poorer overall survival compared to those with low *TNS4* expression levels (Figure 1J, 1K). In concordance with the public dataset results, our data showed that TNS4 protein was significantly overexpressed in HNSCC tissues relative to ANTs (Figure 1L, 1M). Additionally, utilizing the 4-NQO carcinogenesis model, we observed a stepwise elevation in TNS4 expression, transitioning from normal tissues to dysplastic tissues and culminating in tumor tissues (Figure 1N-1P). TNS4 expression was substantially higher in HNSCC cell lines compared to normal epithelial cells, such as NHOK and NHEK. Moreover, TNS4 overexpression was observed in HNSCC cell lines (UM1 and UM5) with greater tumorigenic potential relative to corresponding cell lines with lower tumorigenic potential (UM2 and UM6) (Figure 1Q). The IHC staining results revealed that the staining intensity of TNS4 was significantly higher in HNSCC tissues with advanced TNM stages or lymph node metastasis (LNM) (Figure 1R-1T). Consequently, survival analysis demonstrated that HNSCC patients in the high TNS4 staining group experienced worse overall survival compared to those in the low TNS4 staining group (Figure 1U).

### TNS4 depletion mitigates malignant features of HNSCC cells *in vitro* and *in vivo*

**Figure 2:**
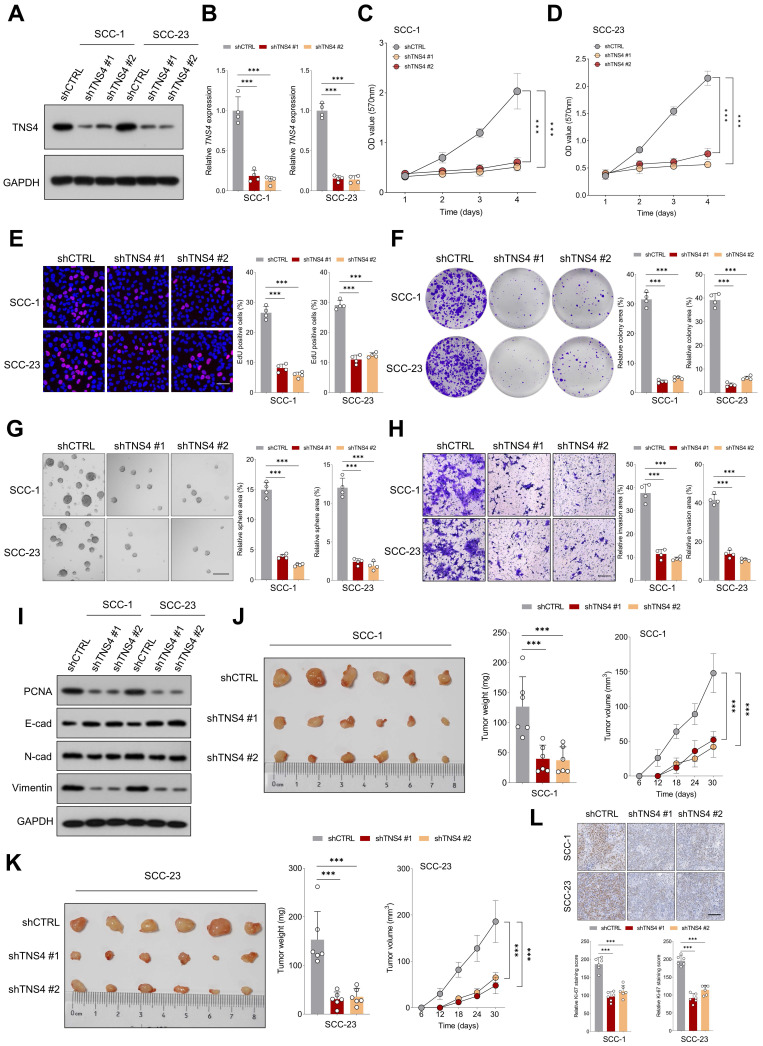
TNS4 depletion mitigates malignant features of HNSCC cells *in vitro* and *in vivo*. (A-B) Western blot and qPCR analysis of TNS4 expression in HNSCC cells subjected to the indicated treatments. (C-D) OD values at specified time points for TNS4-depleted cells and control cells. (E) Percentage of EdU-positive cells in the TNS4-depleted groups versus the control group (scale bar=50 μm). (F-G) Colony and sphere-forming capabilities of TNS4-depleted cells compared to control cells (scale bar=200 μm). (H) Impact of TNS4 depletion on the invasive capacity of HNSCC cells (scale bar=200 μm). (I) Western blot analysis of PCNA, E-cadherin, N-cadherin, and vimentin expression in TNS4-depleted cells versus control cells. (J-K) Comparison of tumor size, tumor weight, and tumor volume between the TNS4-depleted groups and the control group. (L) Staining intensity of Ki-67 in xenograft tissues derived from TNS4-depleted cells versus control cells (scale bar=100 μm).

Lentiviruses carrying shRNAs targeting TNS4 (shTNS4 #1 and shTNS4 #2) were utilized to examine the effects of TNS4 depletion on HNSCC cell lines SCC-1 and SCC-23. The findings indicated a significant decrease in TNS4 expression in cancer cells treated with shTNS4 compared to control cells (Figure 2A, 2B). MTT and EdU assays revealed notable reductions in optical density (OD) values at specified time points and a substantially lower percentage of EdU-positive cells in TNS4-depleted cancer cells (Figure 2C-2E). Colony and tumor sphere formation assays further revealed significantly reduced colony and tumor sphere formation capabilities in HNSCC cells upon TNS4 depletion compared to control cells (Figure 2F, 2G). In line with these results, the matrigel invasion assay demonstrated a diminished invasion capacity in HNSCC cells with TNS4 depletion compared to control cells (Figure 2H). The western blotting analysis revealed that TNS4 depletion led to a reduction in PCNA, N-cadherin, and vimentin expression, while simultaneously enhancing E-cadherin expression (Figure 2I). In a xenograft tumor model, tumors generated by TNS4-depleted SCC-1 cells exhibited considerably smaller size, reduced weight, and decreased volume compared to those produced by control cells (Figure 2J). Comparable observations were made in SCC-23 cells (Figure 2K). Moreover, IHC analysis showed that the staining intensity of Ki-67 was markedly lower in xenograft tumor tissues derived from TNS4-depleted cells than in those originating from control cells (Figure 2L).

### TNS4 overexpression enhances malignant features of HNSCC cells *in vitro* and *in vivo*

We further examined the impact of TNS4 overexpression on the malignancy features of HNSCC cells. Western blot analysis demonstrated a significant upregulation of TNS4 expression in HNSCC cells infected with TNS4-overexpressing lentiviruses (Figure S1A, 1B). Our data revealed that cancer cells overexpressing TNS4 exhibited a marked increase in proliferation compared to control cells, as evidenced by MTT and EdU assays (Figure S1C-1E). Additionally, colony and tumor sphere formation assays showed a substantial enhancement in the relative areas of colonies and spheres in TNS4-overexpressing cancer cells relative to control cells (Figure S1F, 1G). The matrigel invasion assay also indicated that ectopic TNS4 expression promoted the invasiveness of HNSCC cells (Figure S1H). Western blotting analysis demonstrated that TNS4 overexpression augmented the expression levels of PCNA, N-cadherin, and vimentin, while it concurrently suppressed E-cadherin expression (Figure S1I). To substantiate these findings *in vivo*, we utilized a xenograft tumor model and observed that TNS4-overexpressing SCC-1 cells generated tumors with considerably larger size, weight, and volume compared to tumors generated by their control counterparts (Figure S1J). Analogous results were obtained for SCC-23 cells (Figure S1K). Furthermore, the intensity of Ki-67 staining was notably elevated in xenograft tumor tissues derived from TNS4-overexpressing cells relative to those derived from control cells (Figure S1L).

### Importance of TNS4 in maintaining the stability of the integrin α5β1 complex

**Figure 3:**
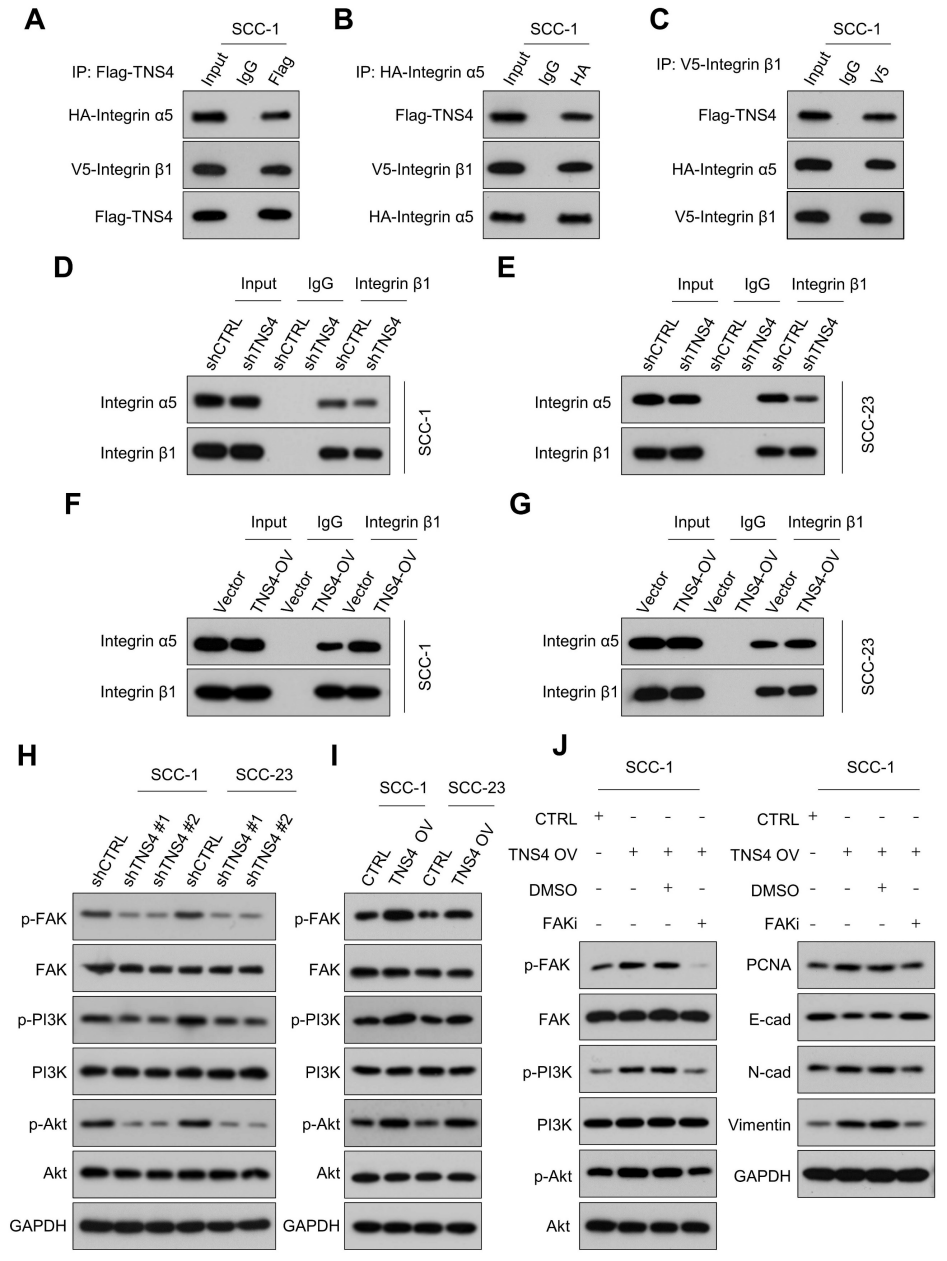
Importance of TNS4 in maintaining the stability of the integrin α5β1 complex and regulating FAK/PI3K/Akt signaling pathway. (A-C) Interactions among TNS4, integrin α5, and integrin β1. (D-E) Impact of TNS4 depletion on the interaction between integrin α5 and integrin β1. (F-G) Influence of TNS4 overexpression on the interaction between integrin α5 and integrin β1. (H-I) Effect of TNS4 depletion or overexpression on the expression levels of p-FAK, FAK, p-PI3K, PI3K, p-Akt, and Akt. (J) Expression of p-FAK, FAK, p-PI3K, PI3K, p-Akt, Akt, PCNA, E-cadherin, N-cadherin, and vimentin in HNSCC cells subjected to indicated treatments.

Previous research has indicated that TNS family members interact with the integrin family to modulate cellular function, and a physical association between TNS4 and integrin β1 has been identified 18, 19. In addition, GSEA revealed consistent enrichment of integrin-related pathways in the *TNS4*-high group within the TCGA HNSCC cohort, suggesting a potential role for TNS4 in modulating integrin-mediated cellular processes in HNSCC (Figure S2). Our Co-IP results verified the interaction between TNS4, integrin β1, and integrin α5 (Figure 3A-3C). Furthermore, the PTB domain of TNS4 was found to physically interact with the cytoplasmic domain of integrin β1 in HNSCC cells (Figure S3A-3D). Interestingly, TNS4 exhibited no interaction with other integrin α subunits (data not shown), leading us to hypothesize that TNS4 might be crucial for the formation of the integrin α5β1 complex. As expected, TNS4 depletion significantly diminished the interaction between integrin β1 and integrin α5 (Figure 3D, 3E), whereas TNS4 overexpression enhanced their association (Figure 3F, 3G). These findings indicate that TNS4 plays a critical role in maintaining integrin α5β1 complex stability in HNSCC cells.

### Critical role of the TNS4-integrin α5β1 axis in modulating FAK/PI3K/Akt signaling pathway in HNSCC

It is well-established that integrin signaling fosters tumor growth via FAK, acting as a key regulator of the PI3K/Akt signaling pathway 20-22. The GSEA further revealed that the FAK pathway, the focal adhesion pathway, and the PI3K-Akt-mTOR signaling pathway were significantly enriched in the *TNS4*-high group within the TCGA HNSCC cohort (Figure S4A, 4B). Accordingly, we investigated the effects of TNS4 depletion or overexpression on the expression of the FAK/PI3K/Akt signaling pathway. Our western blot results showed that TNS4 depletion markedly diminished the expression of p-FAK, p-PI3K, and p-Akt in HNSCC cells, while resulting in minimal changes on the expression of total FAK, PI3K, and Akt (Figure 3H). In contrast, ectopic TNS4 expression led to a significant elevation in the phosphorylation levels of FAK, PI3K, and Akt in HNSCC cells (Figure 3I). Moreover, treatment with a FAK inhibitor (VS-4718) led to a reduction in the levels of p-FAK, p-PI3K, p-Akt, PCNA, N-cadherin, and vimentin, all of which had been elevated due to TNS4 overexpression. Concurrently, the treatment resulted in a significant increase in E-cadherin levels, reversing the downregulation induced by TNS4 upregulation (Figure 3J). These observations underscore the critical role of TNS4 in modulating the FAK/PI3K/Akt signaling pathway. Furthermore, we explored the contribution of the integrin α5β1 complex in the TNS4-mediated activation of the FAK/PI3K/Akt signaling pathway. As expected, the effects of TNS4 overexpression on p-FAK, p-Akt, PCNA, vimentin, and N-cadherin were significantly attenuated by siRNA-mediated silencing of either integrin α5 or β1. This silencing also led to a notable increase in E-cadherin levels, counteracting their downregulation due to TNS4 overexpression (Figure S5A, 5B).

### FAK inhibition counteracts the tumorigenic potential of TNS4-overexpressing HNSCC cells *in vitro* and* in vivo*

**Figure 4:**
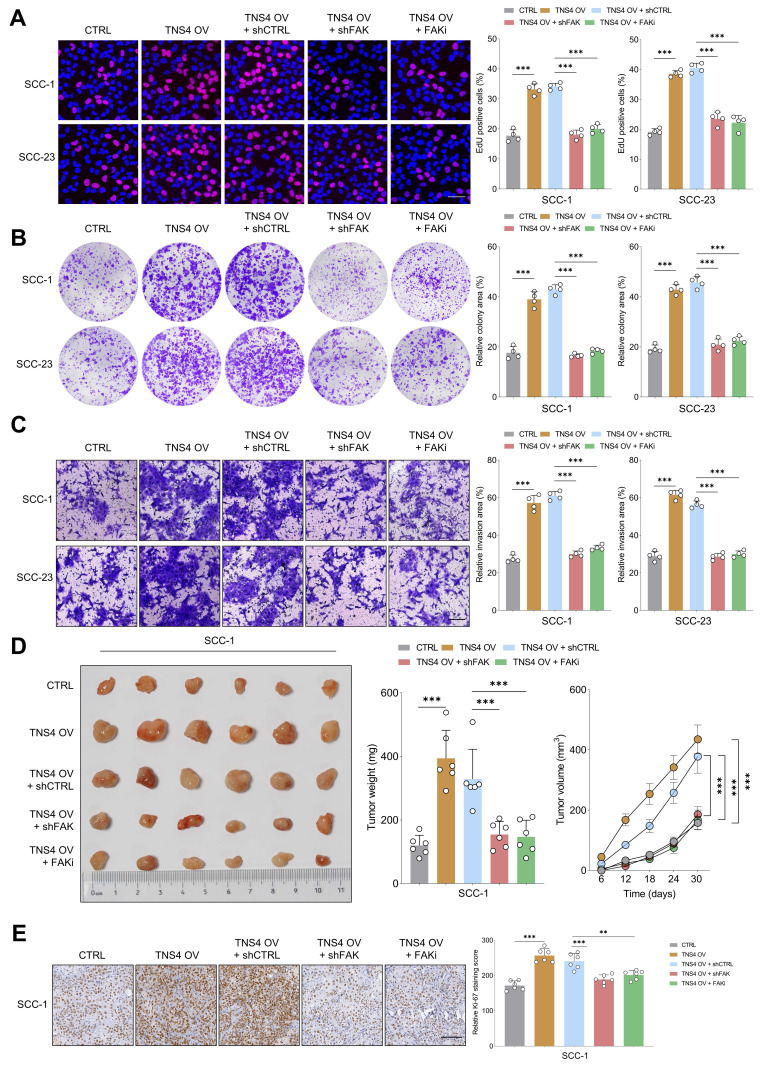
FAK inhibition mitigates the tumorigenic potential of TNS4-overexpressing HNSCC cells *in vitro* and *in vivo*. (A) Percentage of EdU positive cells in groups subjected to the indicated treatments (scale bar=50 μm). (B) Colony formation capacity of HNSCC cells following the indicated modifications. (C) Invasion capacity of cancer cells subjected to the indicated modifications (scale bar=200 μm). (D) Comparison of tumor size, weight, and volume across the indicated groups. (E) Ki-67 staining intensity in xenograft tumor tissues derived from cells subjected to specified treatments (scale bar=100 μm).

Subsequently, we sought to ascertain whether FAK inactivation would mitigate the tumor-promoting effects of the TNS4-integrin α5β1 axis in HNSCC cells. The EdU assay revealed that either shFAK or FAK inhibitor administration significantly diminished the enhanced proliferative capacity of HNSCC cells driven by TNS4 overexpression (Figure 4A). Similarly, colony formation and matrigel invasion assay indicated that FAK suppression partially attenuated the enhanced colony formation and invasive capabilities of HNSCC cells promoted by TNS4 overexpression (Figure 4B, 4C). The xenograft tumor model further confirmed that the increases in tumor size, weight, and volume attributable to TNS4 overexpression were partially mitigated upon treatment with either shFAK or a FAK inhibitor (Figure 4D). Additionally, the intensity of Ki-67 staining was significantly increased in xenograft tumors derived from cells with TNS4 overexpression, and this elevation was effectively mitigated through the administration of either shFAK or a FAK inhibitor (Figure 4E). Comparable results were obtained *in vivo* with SCC-23 cells, as illustrated in Figure S6A-6D. These findings suggest that the TNS4-integrin α5β1 axis plays a crucial role in activating the FAK-mediated signaling pathway, and FAK deactivation effectively attenuates the tumor-promoting effect of TNS4 overexpression in HNSCC cells.

### TNS4-mediated FAK activation promotes EMT and metastasis in HNSCC cells by enhancing the interaction between TGFβRI and TGFβRII

**Figure 5:**
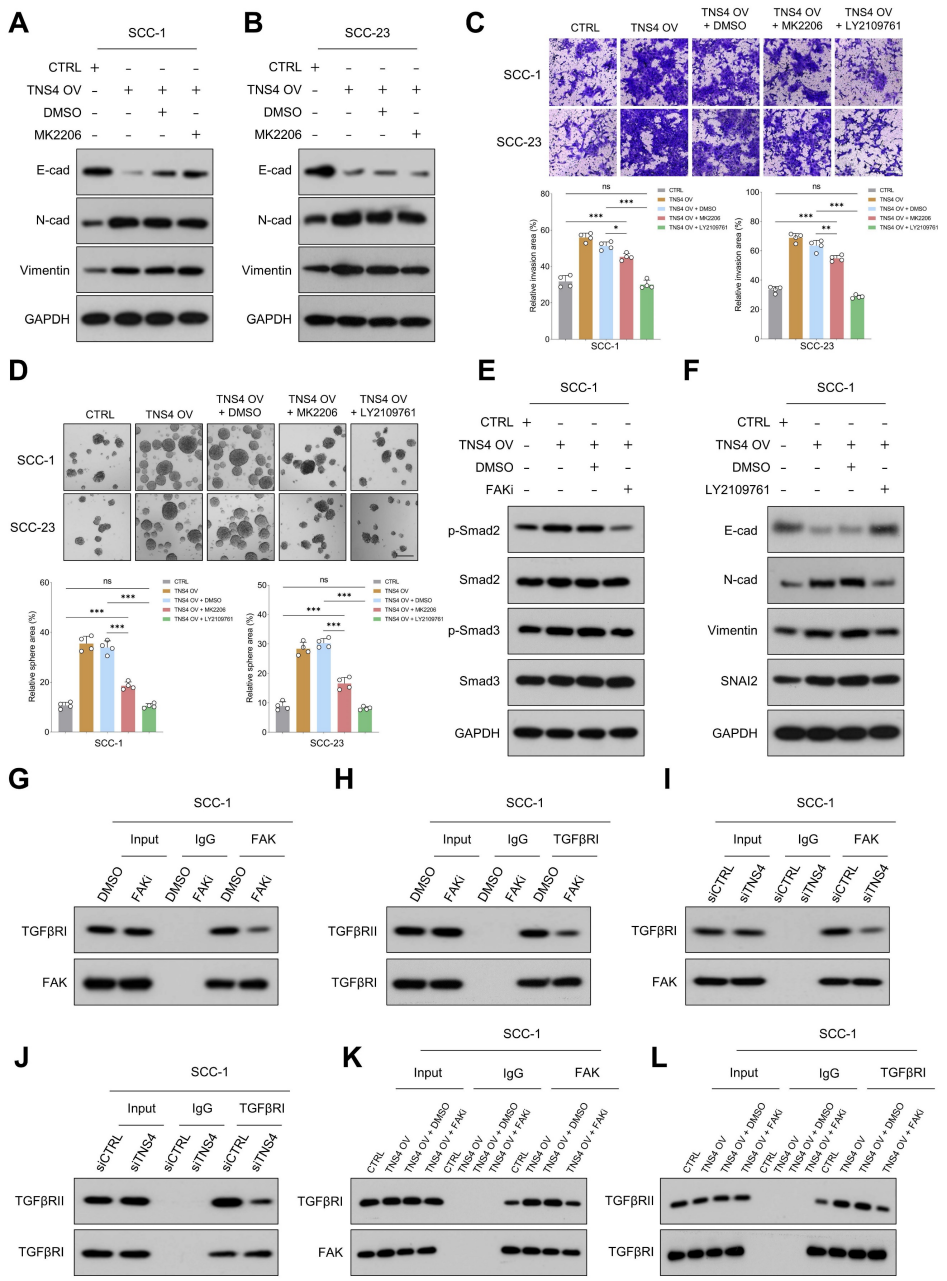
TNS4-facilitated FAK activation enhances EMT and metastasis in HNSCC cells by promoting TGFβRI and TGFβRII interaction. (A-B) Expression levels of E-cadherin, N-cadherin, and vimentin in TNS4-overexpressing cells treated with an Akt inhibitor. (C) Invasion potential of HNSCC cells under indicated treatment conditions (scale bar=200 μm). (D) The sphere-forming abilities of HNSCC cells with indicated modifications (scale bar=200 μm). (E) Western blotting analysis of p-Smad2, Smad3, p-Smad3, and Smad2 expression in HNSCC cells subjected to the indicated modifications. (F) Expression of E-cadherin, N-cadherin, vimentin, and SNAI2 in TNS4-overexpressing cells treated with LY2109761. (G-H) Effects of FAK inhibitor on the interactions between FAK and TGFβRI as well as between TGFβRI and TGFβRII. (I-J) Influence of TNS4 depletion on the interactions between FAK and TGFβRI, and between TGFβRI and TGFβRII. (K-L) Impact of TNS4 overexpression on the interactions between FAK and TGFβRI, and between TGFβRI and TGFβRII.

Our study examined the extent to which Akt inhibition could mitigate the pro-tumorigenic activities associated with TNS4 overexpression, revealing noteworthy findings. Both MTT and western blot assays demonstrated that Akt inhibition significantly reduced the proliferative capacity of HNSCC cells and attenuated the elevated expression of PCNA induced by TNS4 overexpression (Figure S7A-7D). However, the effect of the Akt inhibitor on the expression of EMT-related markers (E-cadherin, N-cadherin, Vimentin), which were altered by TNS4 overexpression, was comparatively modest (Figure 5A, 5B). Furthermore, matrigel invasion assay and tumor sphere-forming assay both revealed that the Akt inhibitor did indeed attenuate the enhanced invasion capacity and sphere-forming ability induced by TNS4 overexpression in HNSCC cells. Nonetheless, the proportion of invading cells, as well as the tumor sphere-forming capacity, remained significantly elevated in the TNS4 OV + Akt inhibitor group relative to the CTRL group (Figure 5C, 5D). Taken together, these findings underscore the crucial role of the FAK/PI3K/Akt pathway in promoting the proliferation and survival of HNSCC cells enhanced by TNS4 overexpression. However, the data also indicate the potential contribution of alternative signaling pathways to the enhancement of EMT processes and metastatic potential in cancer cells influenced by TNS4.

TGFβ signaling, known for its pivotal role in EMT processes and tumor metastasis 23, 24, was found to be enriched in *TNS4*-high tumor tissues across multiple independent HNSCC cohorts (Figure S8). This suggests a robust correlation between TNS4 and TGFβ signaling pathway. The invasive and tumor sphere-forming abilities of HNSCC cells, enhanced by TNS4 overexpression, were significantly attenuated upon treatment with the TGFβ signaling inhibitor LY2109761 (Figure 5C, 5D). Additionally, western blot analysis indicated that the elevated expression levels of p-Smad2 and p-Smad3, induced by TNS4 overexpression, were mitigated by FAK inhibition (Figure 5E, Figure S9A). These findings collectively suggest that TNS4 primarily regulates EMT and metastasis through the FAK-TGFβ axis. As anticipated, LY2109761 dramatically suppressed the TNS4-overexpression-promoted expression of N-cadherin, vimentin, and SNAI2 in HNSCC cells. Concurrently, it substantially upregulated the levels of E-cadherin, which had been suppressed by TNS4 overexpression (Figure 5F, Figure S9B). The intricate relationship between TNS4 and the TGFβ signaling pathway was further investigated. Depletion of TNS4 resulted in reduced expression of critical elements within the TGFβ signaling cascade. Notably, this downregulation was substantially reversed upon the addition of recombinant TGFβ (Figure S10A). Conversely, TNS4 overexpression led to an increase in the expression levels of these pivotal signaling components, an effect that was further potentiated by the addition of recombinant TGFβ (Figure S10B). We also assessed the influence of TGFβ activation on TNS4 expression. Our findings suggest that the exogenous administration of TGFβ exerted a minimal effect on TNS4 expression levels, irrespective of variations in exposure time and dosage (Figure S11A, 11B). These observations suggest that TNS4 functions as an upstream modulator in the TGFβ signaling pathway, thereby influencing its downstream elements.

Previous research has established that FAK modulates TGFβ signaling by influencing the interaction between TGFβRI and TGFβRII 25. Co-IP results revealed that FAK inhibition significantly reduced not only the interaction between FAK and TGFβRI (Figure 5G, Figure S12A), but also the interaction between TGFβRI and TGFβRII (Figure 5H, Figure S12B). Subsequently, we assessed the impact of TNS4 modulation on the interactions among FAK, TGFβRI, and TGFβRII. The results indicated that siTNS4 attenuated the interaction between FAK and TGFβRI (Figure 5I, Figure S12C), as well as the interaction between TGFβRI and TGFβRII (Figure 5J, Figure S12D). In contrast, TNS4 overexpression augmented these interactions. Notably, these enhancements were counteracted upon administration of FAKi (Figure 5K, 5L, Figure S12E, 12F). Furthermore, we showed that FAK inhibitor treatment led to a significant reduction in tyrosine phosphorylation on TGFβRI (Figure S13). Together, these data suggest that TNS4-mediated FAK activation promotes EMT and HNSCC cell metastasis by enhancing the TGFβ signaling pathway.

### TNS4 is upregulated under the hypoxia microenvironment and transcriptionally regulated by HIF-1α

**Figure 6:**
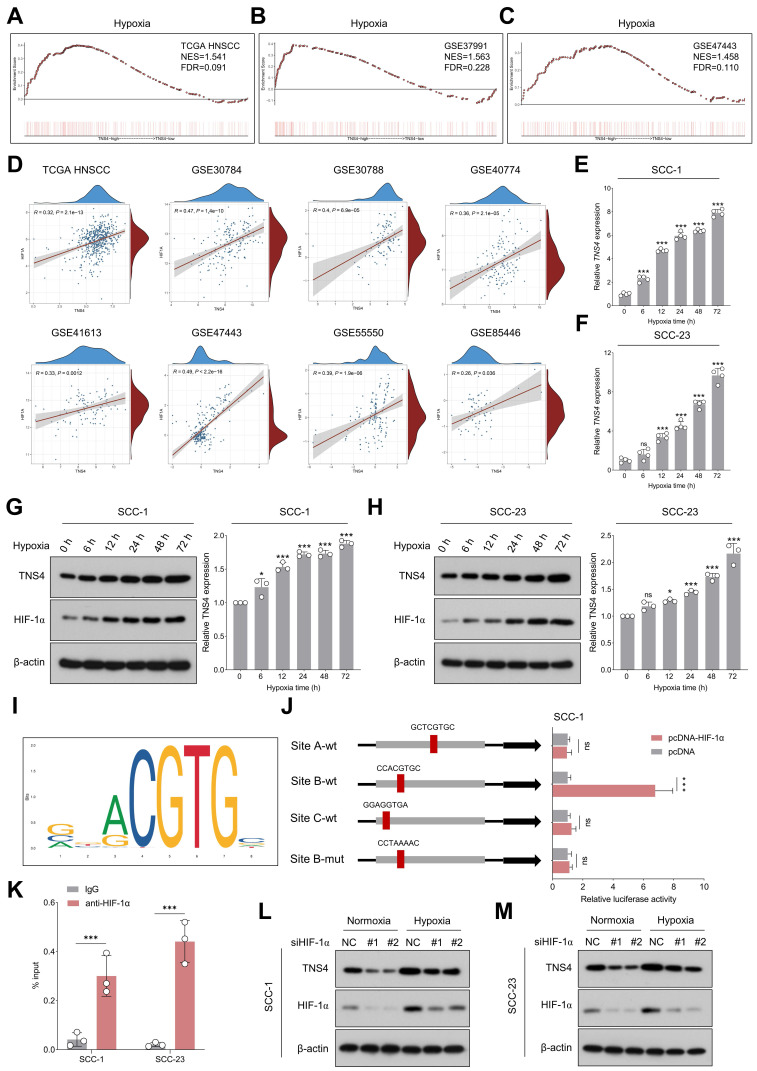
TNS4 is upregulated under the hypoxia microenvironment and transcriptionally regulated by HIF-1α. (A-C) GSEA reveals enrichment of the hypoxia signature in *TNS4*-high HNSCC tissues in TCGA HNSCC, GSE37991, and GSE47443. (D) Correlation between *HIF1A* and *TNS4* expression across multiple independent HNSCC cohorts. (E-F) Temporal dynamics of *TNS4* mRNA expression under hypoxic conditions at indicated time points. (G-H) Temporal dynamics of TNS4 protein expression under hypoxic conditions at indicated time points. (I) HIF-1α binding motif as identified by the JASPAR database. (J) Relative luciferase activities in SCC-1 cells subjected to indicated treatments. (K) ChIP-qPCR analysis showing the enrichment of HIF-1α at the *TNS4* promoter region. (L-M) Impact of HIF-1α depletion on TNS4 expression in HNSCC cells under normoxic and hypoxic conditions.

Having elucidated the downstream pathways influenced by TNS4, we next identified potential upstream regulators that could be modulating TNS4 expression in HNSCC. GSEA analysis indicated significant enrichment of hypoxia-related gene signatures in the high *TNS4* expression group within the TCGA HNSCC cohort, a finding that was further validated by data from the GSE37991 and GSE47443 datasets (Figure 6A-6C). In addition, a strong positive correlation between *HIF1A* and *TNS4* was consistently observed across multiple independent HNSCC cohorts, including TCGA HNSCC, GSE30784, GSE30788, GSE40774, GSE41613, GSE47443, GSE55550, and GSE85446 (Figure 6D).

As shown in Figure 6E-6H, exposure to hypoxic conditions led to a progressive elevation in both TNS4 mRNA and protein levels in HNSCC cells. However, neither overexpression nor depletion of TNS4 significantly affected HIF-1α expression under normoxia or hypoxia conditions (Figure S14A-14H), suggesting HIF-1α as a potential upstream TNS4 regulator. The JASPAR database-predicted HIF-1α binding motif is depicted in Figure 6I. To elucidate the role of HIF-1α in the transcriptional regulation of *TNS4*, we synthesized both wild-type and mutant luciferase reporter vectors harboring putative HIF-1α-binding sites within the *TNS4* promoter region. Our luciferase reporter assays indicated that HIF-1α overexpression significantly elevated the transcriptional activity of *TNS4* at Site B (CCACGTGC). Importantly, this elevation was abolished when the nucleotide sequence at Site B was altered to CCTAAAAC, as depicted in Figure 6J. ChIP-qPCR further validated the enrichment of HIF-1α at the *TNS4* promoter region (Figure 6K). Consistently, silencing of HIF-1α led to a significant reduction in TNS4 expression levels in HNSCC cells, under both normoxic and hypoxic conditions (Figure 6L, 6M).

### The clinical significance of HIF-1α/TNS4/p-FAK axis in HNSCC

**Figure 7:**
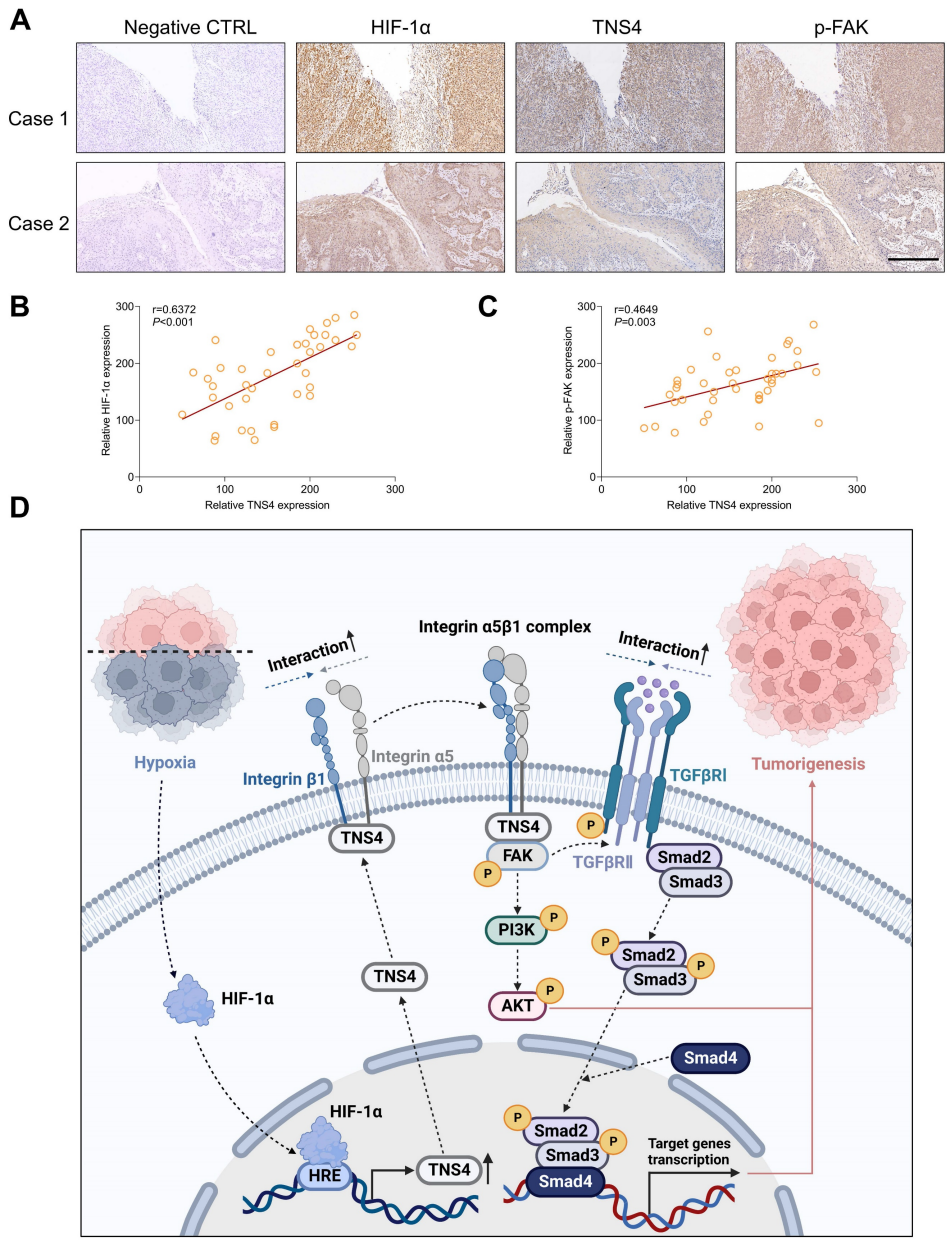
Clinical relevance of the HIF-1α/TNS4/p-FAK axis in HNSCC. (A) Representative IHC images demonstrating HIF-1α, TNS4, and p-FAK staining in HNSCC cases (scale bar=200 μm). (B) Correlation analysis of staining intensities between HIF-1α and TNS4 in HNSCC. (C) Analysis of the correlation between the staining intensities of TNS4 and p-FAK in HNSCC. (D) Schematic representation of the proposed mechanism underlying the TNS4-promoted progression of HNSCC.

We subsequently explored the clinical significance of the HIF-1α/TNS4/p-FAK axis using HNSCC specimens from our in-house cohort. Consistent with the results obtained from public datasets, our IHC analysis revealed a positive correlation between the expression of HIF-1α and TNS4 proteins (Figure 7A, 7B). Furthermore, a positive correlation was observed between the expression of TNS4 and p-FAK proteins in HNSCC tissues (Figure 7C). These findings suggest that the HIF-1α/TNS4/p-FAK axis plays a crucial role in HNSCC tumorigenesis and is associated with a poorer prognosis for HNSCC patients.

## Discussion

In this study, we report a marked elevation in TNS4 expression in HNSCC tissues, which is strongly associated with poor clinical outcomes. As depicted in Figure 7D, our results demonstrate that inhibiting TNS4 effectively mitigates the oncogenic features of HNSCC cells by attenuating the FAK-mediated Akt and TGFβ signaling pathways. This attenuation occurs *via* the disruption of integrin α5 and integrin β1 interaction. Furthermore, the transcriptional regulation of TNS4 by HIF-1α highlights its pivotal role in the pathogenesis of HNSCC. Therefore, targeted inhibition of TNS4 might be a potentially innovative therapeutic strategy that could improve prognosis and survival rates for patients with HNSCC.

Our study provides novel insights into TNS4's significant upregulation across independent HNSCC cohorts. Elevated levels of TNS4 are strongly correlated with adverse clinical outcomes and increased tumorigenic potential, thereby implicating TNS4 in HNSCC pathogenesis and underscoring its potential as a prognostic indicator. Importantly, this corroborates previous studies that have identified TNS4 overexpression in a variety of other cancers. For instance, in gastric cancer, elevated TNS4 levels were significantly associated with poorer tumor grades, metastatic behavior, and less favorable prognoses 26. In breast cancer, and specifically in highly aggressive and invasive inflammatory breast cancer, TNS4 overexpression was a notable feature 27. Furthermore, increased TNS4 expression correlated with reduced survival rates in primary melanoma 28. Collectively, these findings underscore the clinical importance of TNS4 as a prospective therapeutic target across a spectrum of cancers.

Functional assays underscore TNS4's pivotal role in sustaining HNSCC cells' proliferation and metastatic potential. In non-malignant settings, TNS4 also modulates proliferation. For instance, TNS4 silencing inhibits the proliferation of non-malignant prostate epithelial cells, while TNS4 knockout suppresses keratinocyte proliferation. However, TNS4 overexpression does not typically stimulate further cell proliferation in most cell lines 29. In addition, TNS4 plays a critical role in regulating epithelial sheet invasion and tubulogenesis, as its upregulation is associated with the formation of cellular extensions and tubules and also marks invasive cells within the epithelial sheet 30. Thus, the balanced expression of TNS4 is essential for the proliferation, invasion, and development of normal cells, and its aberrant upregulation could play a pivotal role in promoting the malignant behaviors of cancer cells. For instance, Muharram et al demonstrated the critical interplay between TNS4 and phosphorylated MET, which contributed to cell survival, proliferation, and migration 19. A specific SMARCA4 mutation found in CRC significantly upregulates TNS4 transcription, contributing to enhanced proliferation of CRC cells and patient-derived tumor organoids 31. Moreover, TNS4 played a key role in facilitating metastasis through its interaction with integrin-linked kinase, thereby enhancing tumor invasiveness and leading to poor prognosis in patients with CRC 11.

In our study, we elucidate a novel mechanism whereby TNS4 augments FAK-mediated Akt and TGFβ signaling pathways through enhancing the interaction of integrin α5 and integrin β1. TNS4, a scaffolding protein present at focal adhesions, is known to participate in integrin-mediated signaling 17, 32. Our findings confirm that TNS4 interacts with integrin β1 in HNSCC cells, a consistent observation with previous studies 33. Intriguingly, TNS4 also associates with integrin α5, suggesting its potential role in facilitating the formation of the integrin α5β1 complex, a crucial regulator of cell proliferation, differentiation, angiogenesis, and fibrosis 34, 35. We demonstrate that TNS4 indeed plays a critical role in modulating the interactions between integrin α5 and integrin β1. This complex is implicated in cancer progression predominantly through the FAK/Akt signaling pathways 36. FAK, a regulator of integrin-mediated signaling, coordinates various physiological processes such as migration, invasion, angiogenesis, cell survival, and EMT 37, 38. Our data reveal that the inhibition of FAK significantly curtails the tumor-promoting effects of TNS4 overexpression, underscoring the centrality of FAK activation by the integrin α5β1 complex in TNS4-mediated signaling.

Our observations reveal that the enhanced proliferation observed in TNS4-overexpressing HNSCC cells is entirely abrogated by Akt inhibition, although this intervention has a negligible impact on EMT markers' expression and invasive capability of these cancer cells. These observations indicate that the TNS4 protein governs cellular proliferation and survival in cancerous cells via the FAK/PI3K/Akt signaling pathway. However, the evidence also suggests the involvement of additional pathways in the metastatic capacity enhanced by TNS4 overexpression. In light of its established role in EMT and cancer metastasis, the TGFβ signaling pathway is of particular interest. Our data corroborate prior findings, illustrating the crucial role of FAK in mediating the interaction between TGFβRI and TGFβRII within HNSCC cells, thereby activating downstream Smad-dependent pathways. Importantly, our experimental data reveal that TNS4 plays a pivotal role in mediating the interactions between FAK and TGFβRI, as well as between TGFβRI and TGFβRII. These findings offer new insights into the upstream regulation of the FAK-TGFβ signaling pathway in HNSCC, thereby expanding our understanding of its functional mechanisms.

Recent advances highlight the critical role of hypoxia in promoting malignant traits and therapeutic resistance in HNSCC. Hypoxia, primarily driven by HIF-1α, is known to downregulate ATP6V1A, disrupting lysosomal homeostasis and facilitating the secretion of extracellular vesicles that promote HNSCC progression 39. Furthermore, hypoxia has been shown to contribute to resistance against PD-1 blockade in recurrent or metastatic HNSCC through mechanisms involving immunosuppression and metabolic reprogramming 40. Our results contribute to these findings by demonstrating that hypoxic conditions trigger a HIF-1α-mediated upregulation of TNS4, proposing it as a potential therapeutic target for HNSCC. In the context of HNSCC, characterized by hypoxia due to rapid tumor growth and impaired blood flow, this upregulation of TNS4 in hypoxic microenvironments could explain its commonly observed overexpression in tumor tissues across different cohorts. The depletion of TNS4 has been shown to significantly hinder HNSCC progression, underscoring the pivotal role TNS4 and its downstream signaling pathways play in mediating the oncogenic effects of HIF-1α. While the development of HIF-1α inhibitors has been a key area of research focus, their use has been limited due to issues related to specificity, toxicity, and the complexity of the hypoxia response. Given these limitations, the identification and targeting of TNS4 may provide a promising, more effective, and safer therapeutic strategy for HNSCC.

Under normoxia, HIF-1α is traditionally considered to be rapidly degraded via the ubiquitin-proteasome pathway, making it unavailable for transcriptional activity 41. Therefore, it's generally not expected to directly regulate TNS4 transcription under these conditions. However, our data indicating that HIF-1α depletion affects TNS4 expression even in normoxia suggests a more intricate regulatory relationship. There are several possible reasons for this observation. Firstly, even at low levels, HIF-1α could still exert a significant functional impact. Some gene promoters are extraordinarily sensitive to minimal amounts of a transcription factor, allowing HIF-1α to potentially bind cooperatively with other proteins and initiate TNS4 transcription. Secondly, HIF-1α might play non-canonical roles, possibly acting as a cofactor for other transcriptional regulators or participating in alternative signaling pathways that indirectly affect TNS4 expression. Lastly, the influence of HIF-1α on TNS4 could be mediated indirectly *via* another regulatory gene; in this scenario, HIF-1α would modulate the expression or activity of an intermediary molecule that, in turn, governs TNS4 levels. Further study is needed to unravel the intricate relationship between HIF-1α and TNS4 under different oxygen conditions.

While our study provides compelling evidence suggesting that TNS4 targeting could serve as an effective approach for mitigating HNSCC progression, it is not without limitations. Firstly, despite the promising data, there are currently no small-molecule inhibitors specifically designed to target TNS4. Secondly, the complexity of transcriptomic, proteomic, and metabolomic alterations arising from either the depletion or overexpression of TNS4 remains incompletely understood. Adopting multi-omics strategies could further unravel hitherto unexplored mechanisms through which TNS4 promotes tumorigenesis in HNSCC.

## Conclusion

In summary, our study reveals that TNS4, under the regulation of HIF1-α, contributes to HNSCC tumorigenesis by augmenting FAK-mediated Akt and TGFβ signaling pathways. This enhancement is achieved through the facilitation of interactions between integrin α5 and integrin β1. Our research offers new insights into the complex molecular mechanisms that drive HNSCC tumorigenesis. Importantly, the therapeutic targeting of TNS4 represents a promising strategy for the effective management of HNSCC.

## Supplementary Material

Supplementary figures and tables.

## Abbreviations

HNSCC: head and neck squamous cell carcinoma
TNS4: tensin 4
FAK: focal adhesion kinase
EMT: epithelial-mesenchymal transition
HIF-1α: hypoxia inducible factor 1α
TCGA: The Cancer Genome Atlas
4NQO: 4-nitroquinoline 1-oxide
TGFβ: transforming growth factor-β
