# Supplementary material for: Hypoxia-driven TNS4 fosters HNSCC tumorigenesis by stabilizing integrin α5β1 complex and triggering FAK-mediated Akt and TGFβ signaling pathways
Source: Int J Biol Sci. 2024 Jan 1;20(1):231–48. doi: 10.7150/ijbs.86317 (PMC10750279; doi:10.7150/ijbs.86317)
Supplement: Supplementary file 1 — Supplementary figures and tables. [file ijbsv20p0231s1.pdf]

Supporting Information

Hypoxia-driven TNS4 fosters HNSCC tumorigenesis by stabilizing integrin α5β1 complex and triggering FAK-mediated Akt and TGFβ signaling pathways

Xinyuan Zhao[1,2], Zizhao Mai[2], Liu Liu[1], Ye Lu[2], Li Cui[2#], Jinhua Yu[1#]

1 Jiangsu Key Laboratory of Oral Diseases, Affiliated Hospital of Stomatology, Nanjing Medical University, Nanjing, 210029, China.

2 Stomatological Hospital, School of Stomatology, Southern Medical University, Guangzhou, 510280, China.

[#]Please address the correspondence to

Jinhua Yu, Email: yujinhua@njmu.edu.cn

Li Cui, Email: licui@smu.edu.cn

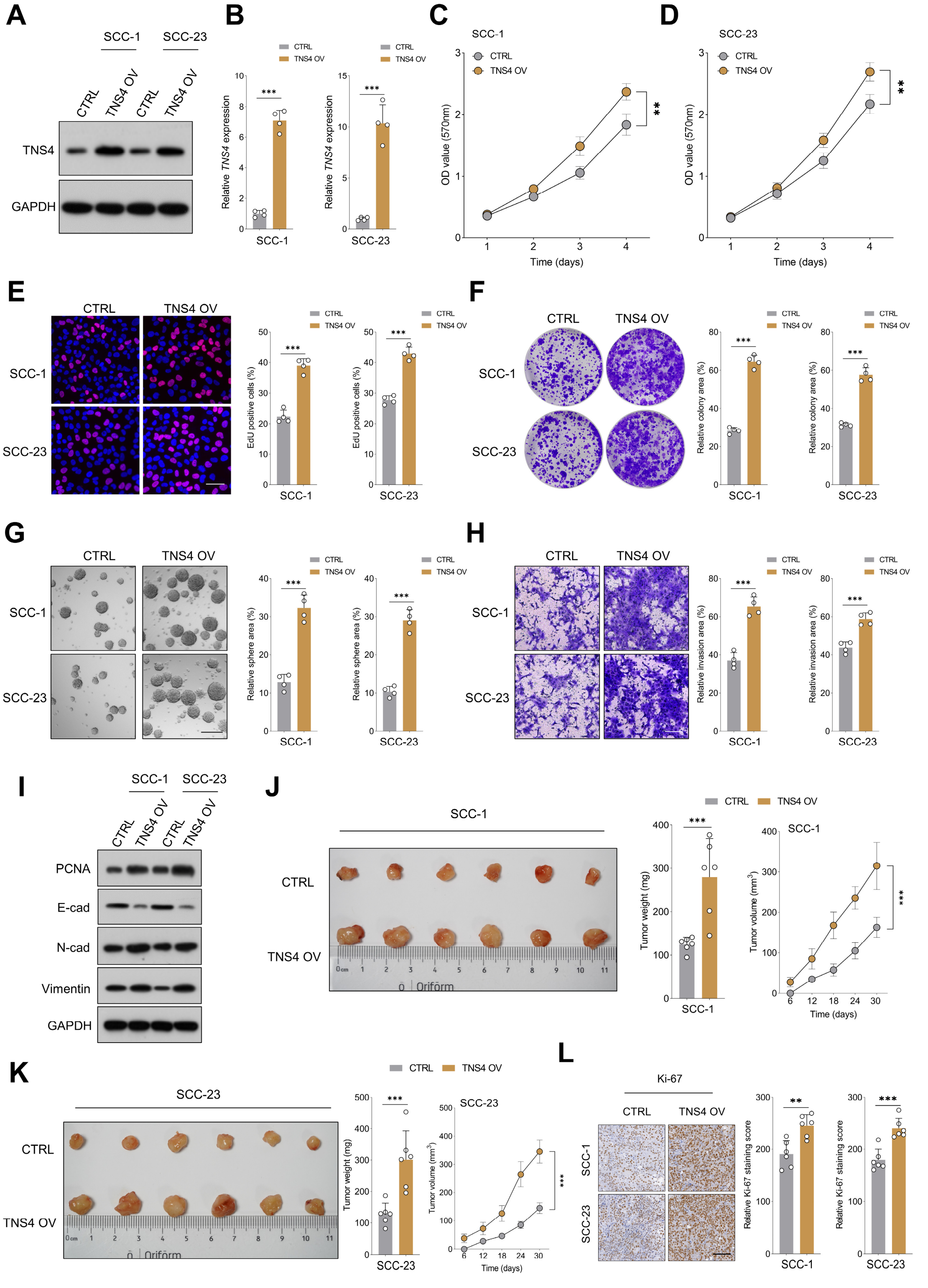

Figure S1. TNS4 overexpression enhances malignant features of HNSCC cells *in vitro* and *in vivo*. (A-B) Validation of TNS4 overexpression in HNSCC cells *via* western blotting and qPCR following specific treatments. (C-D) Measurement of OD values at different time intervals for cells with enforced TNS4 expression and their control

counterparts. (E) Assessment of the proportion of EdU-positive cells in TNS4-overexpressing group versus control group (scale bar=50 μm). (F-G) Comparative analysis of the colony and sphere formation capabilities in cells with TNS4 overexpression and control cells (scale bar=200 μm). (H) Examination of the invasion potential of HNSCC cells following TNS4 overexpression (scale bar=200 μm). (I) Western blot analysis to assess PCNA, E-cadherin, N-cadherin, and vimentin levels in cells with elevated TNS4 expression and control cells. (J-K) Comparison of tumor size, weight, and volume between the group overexpressing TNS4 and the control group. (L) Comparative Ki-67 staining intensity in xenograft tissues generated from cells with TNS4 overexpression and control cells (scale bar=100 μm).

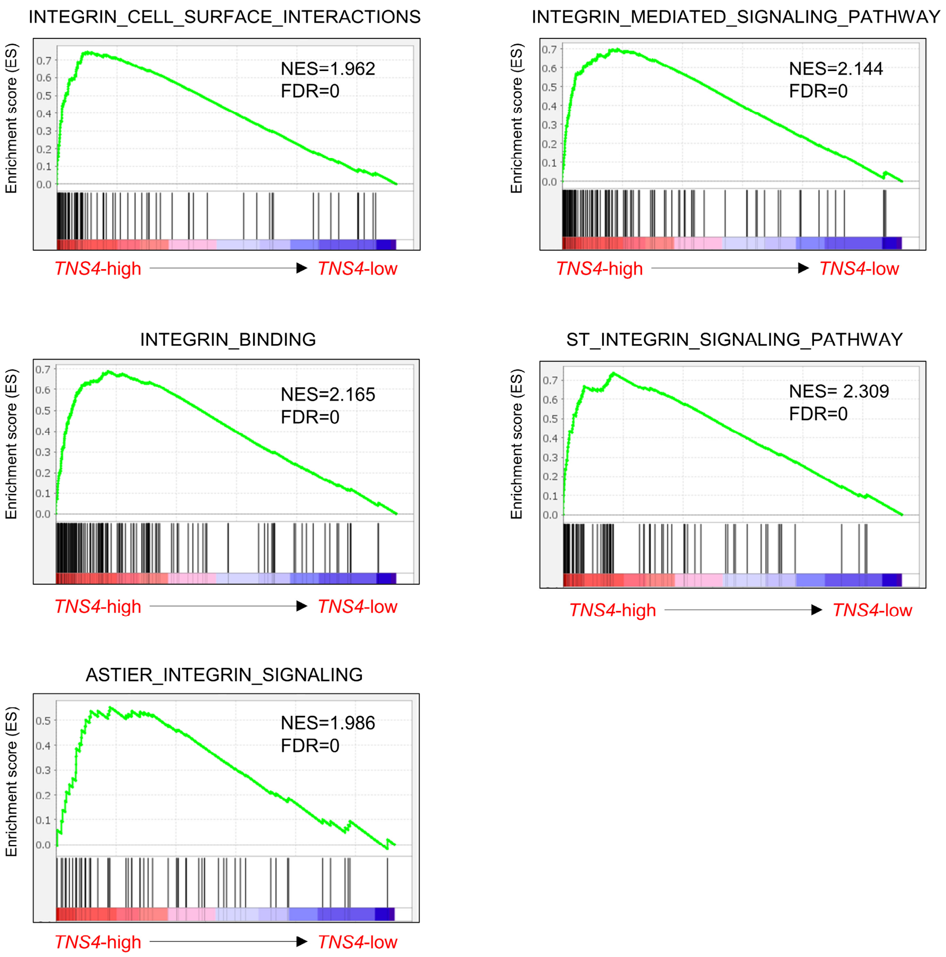

Figure S2. Significant enrichment of integrin-related pathways in the *TNS4*-high subgroup from the TCGA HNSCC cohort.

.

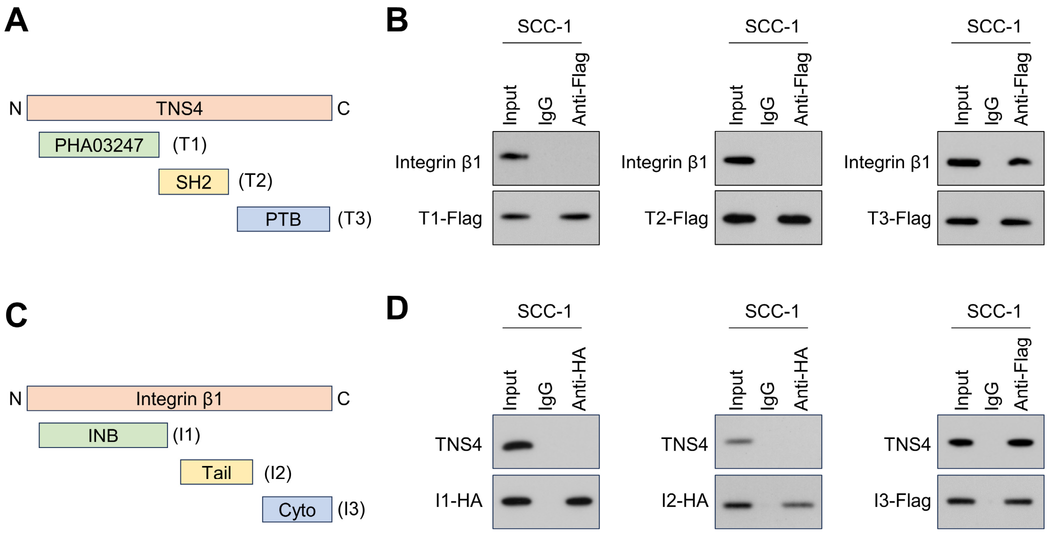

Figure S3. The PTB domain of TNS4 physically interacts with the cytoplasmic domain of integrin β1 in HNSCC cells. (A) Schematic representation of TNS4 domains. (B) Interactions between TNS4 domains and integrin β1 in HNSCC cells as revealed by Co-IP assays. (C) Schematic diagram depicting the domain structure of integrin β1. (D) Interactions between integrin β1domains and TNS4 in HNSCC cells as revealed by Co-IP assays.

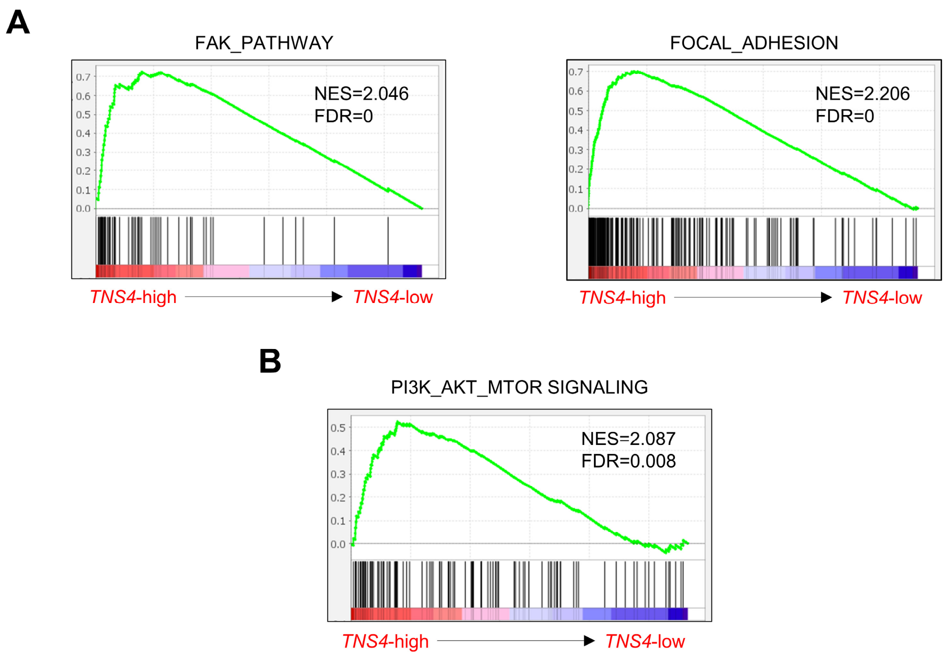

Figure S4. Significant enrichment of FAK pathway, focal adhesion pathway, and PI3K-AKT-mTOR pathway in the *TNS4*-high subgroup from the TCGA HNSCC cohort.

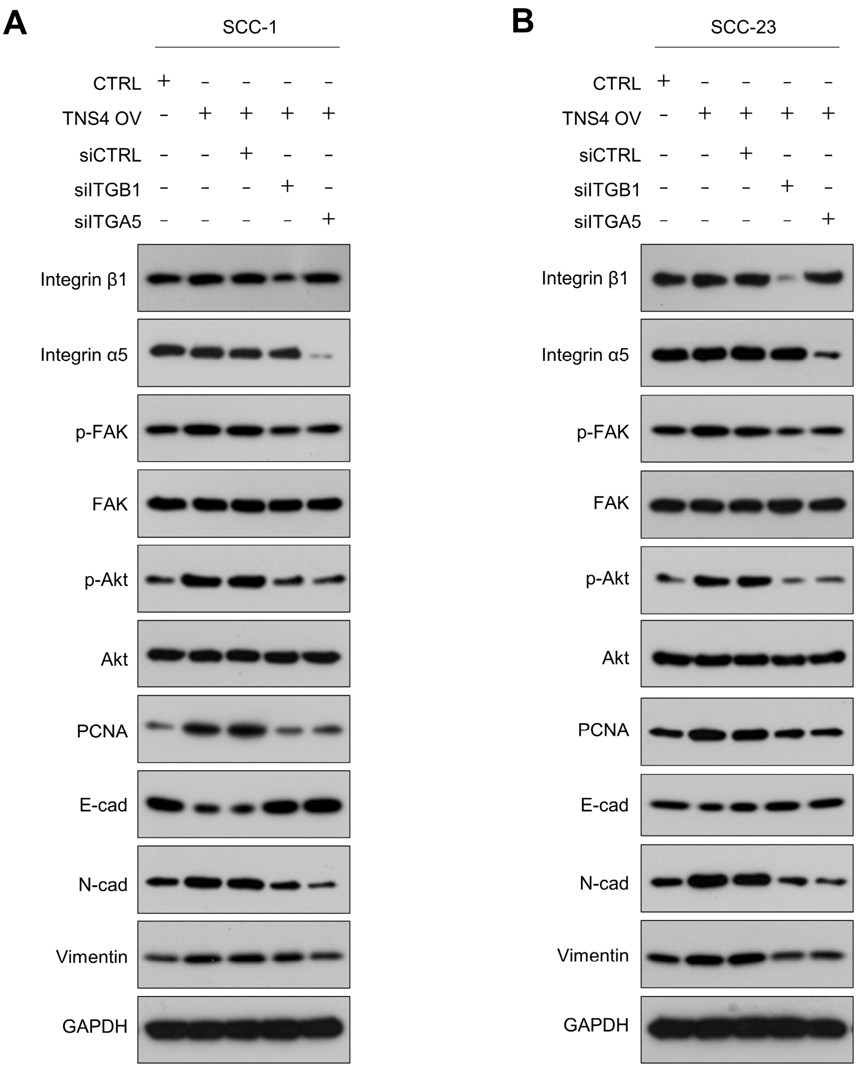

Figure S5. The effects of integrin α5 or integrin β1 depletion on indicated protein expression in TNS4-overexpressing cells. (A-B) Evaluation of protein levels for integrin β1, integrin α5, p-FAK, FAK, p-Akt, Akt, PCNA, E-cadherin, N-cadherin, and vimentin in SCC-1 and SCC-23 cells subjected to specified treatments.

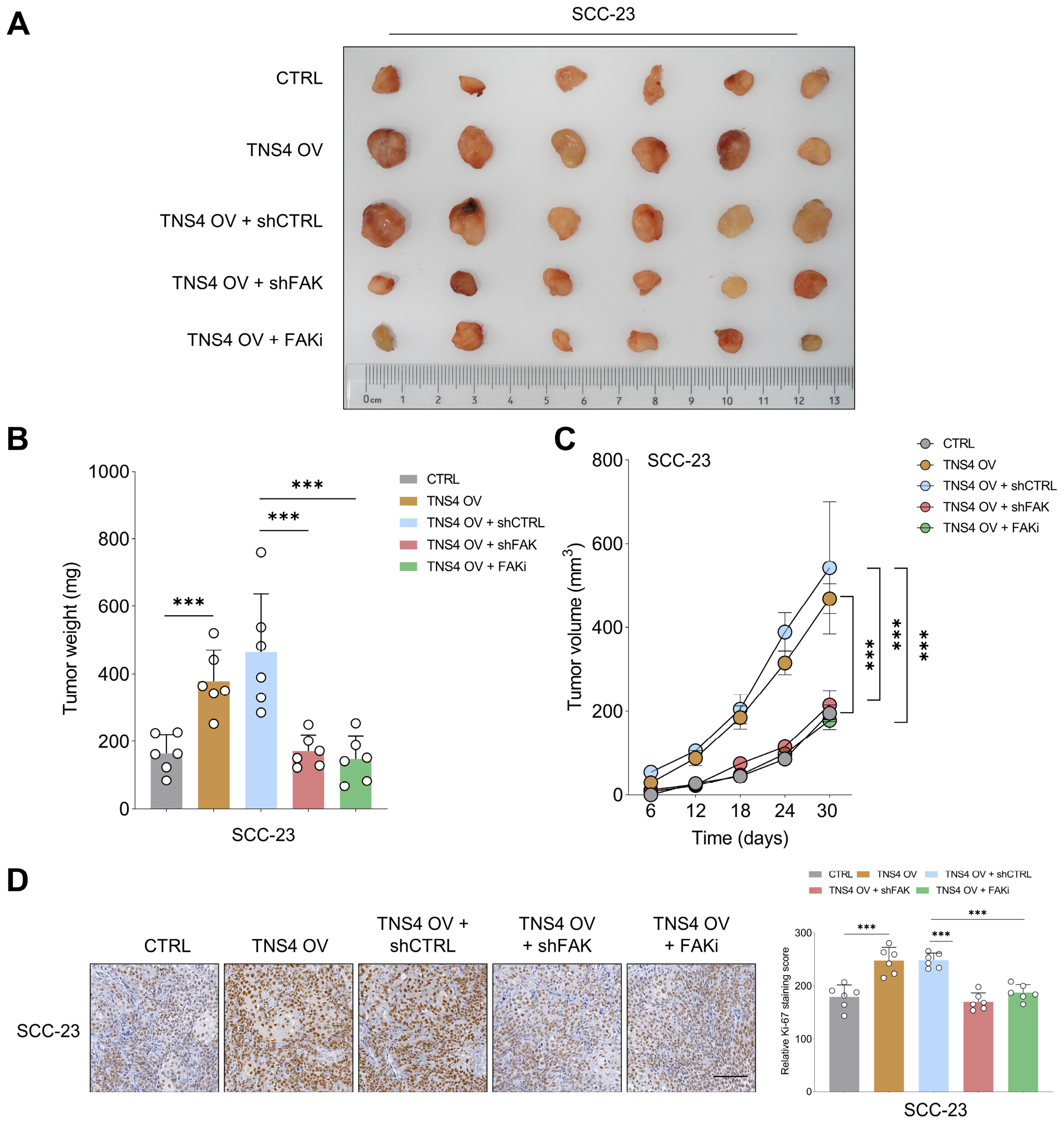

Figure S6. Reduction of tumorigenic potential in TNS4-overexpressing SCC-23 cells through FAK inhibition *in vivo*. (A-C) Comparative analysis of tumor size, weight, and volume among different treatment groups. (D) Evaluation of Ki-67 staining intensity in xenograft tumor tissues derived from SCC-23 cells subjected to specified treatments (scale bar=100 μm).

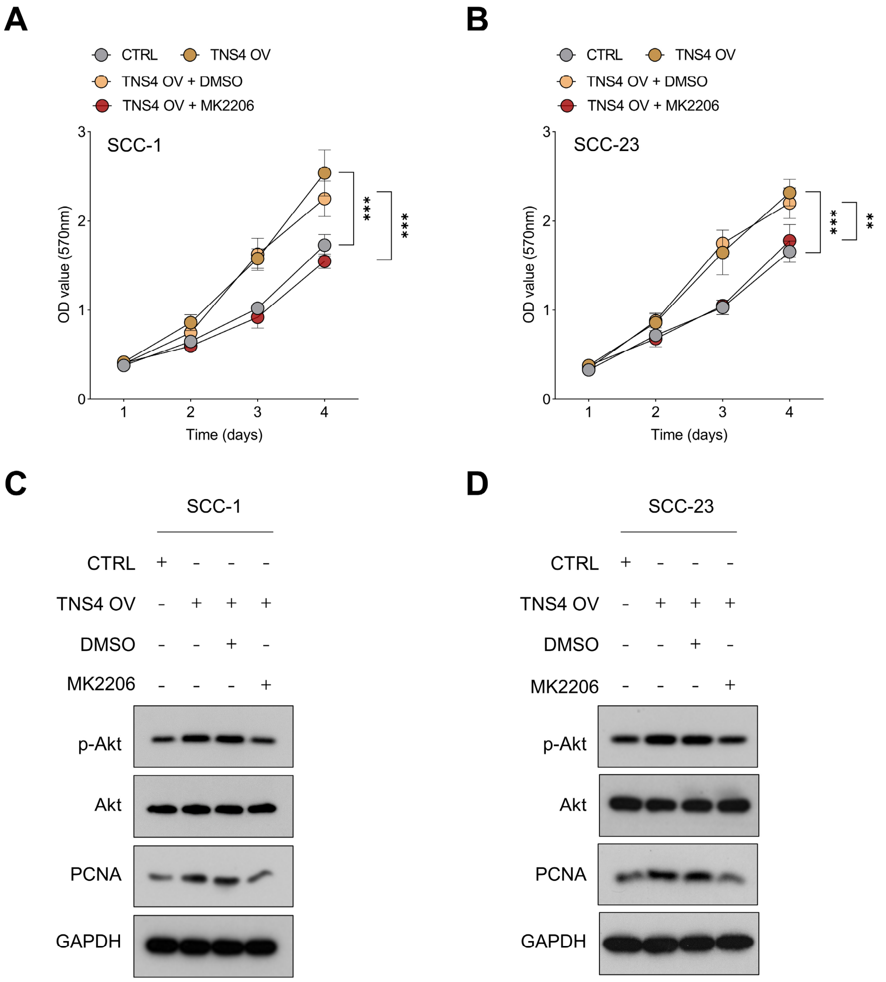

Figure S7. Akt inhibition effectively counteracts TNS4 overexpression-enhanced proliferation in HNSCC cells. (A-B) MTT assay demonstrating the proliferative capacity of HNSCC cells subjected to specified treatments. (C-D) Expression levels of p-Akt, Akt, and PCNA in SCC-1 and SCC-23 cells following respective modifications.

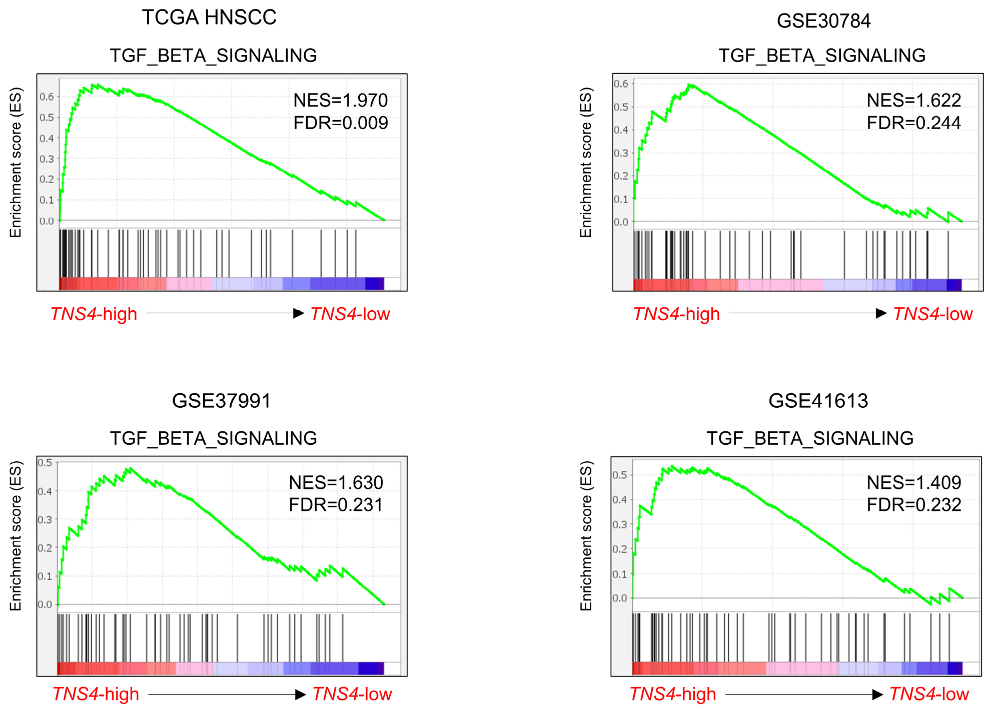

Figure S8. GSEA analysis revealing enrichment of the TGFβ signaling signature in high-*TNS4*-expressing HNSCC tumors across multiple independent cohorts, including TCGA HNSCC, GSE30784, GSE37991, and GSE41613.

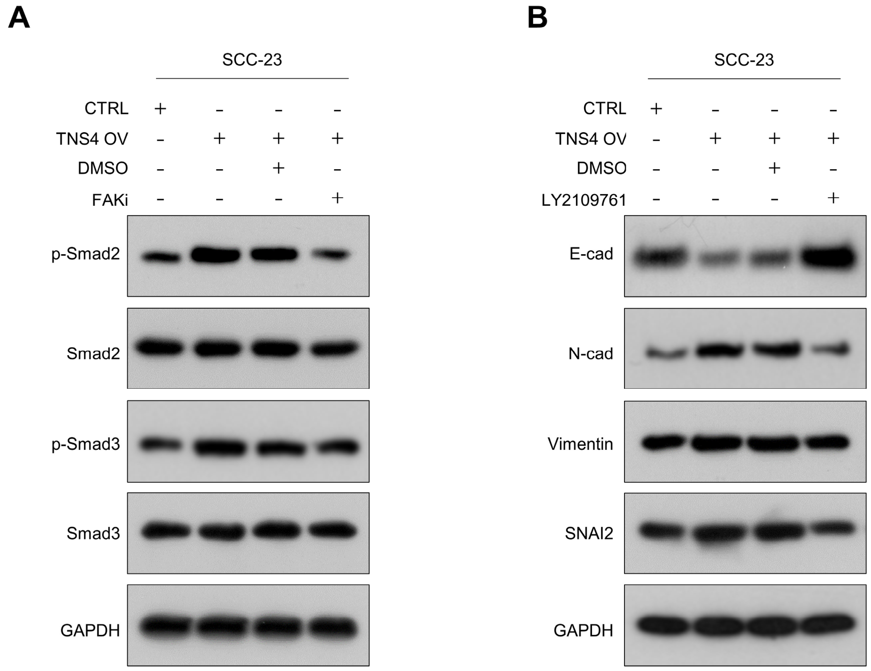

Figure S9. Alterations in TGFβ signaling and EMT marker expression in TNS4-overexpressing SCC-23 cells with indicated treatments. (A) Western blot analysis assessing the expression levels of p-Smad2, Smad3, p-Smad3, and Smad2 in SCC-23 cells subjected to the indicated modifications. (B) Assessment of E-cadherin, N-cadherin, vimentin, and SNAI2 expression levels in TNS4-overexpressing cells following treatment with LY2109761.

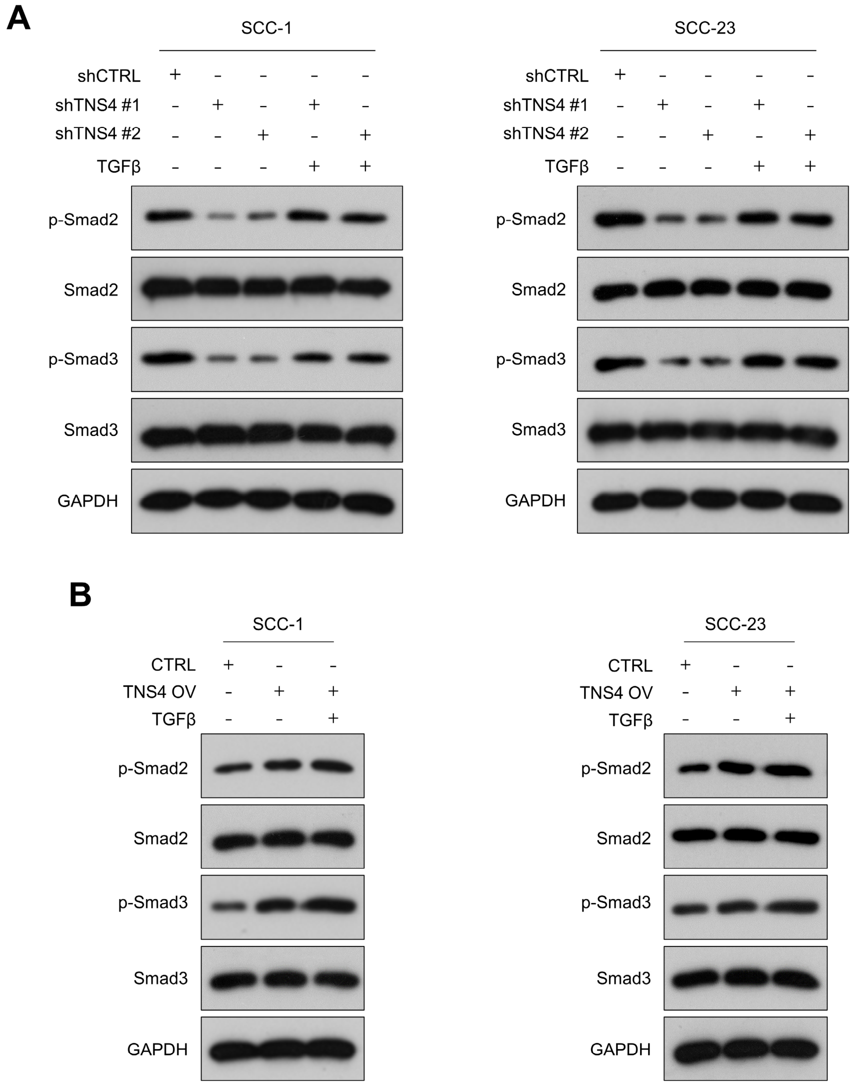

Figure S10. Impact of TNS4 depletion or overexpression on key components of TGFβ signaling in HNSCC cells, with or without TGFβ supplementation. (A-B) Assessment of p-Smad2, Smad2, p-Smad3, and Smad3 expression levels in SCC-1 and SCC-23 cells following the indicated treatments.

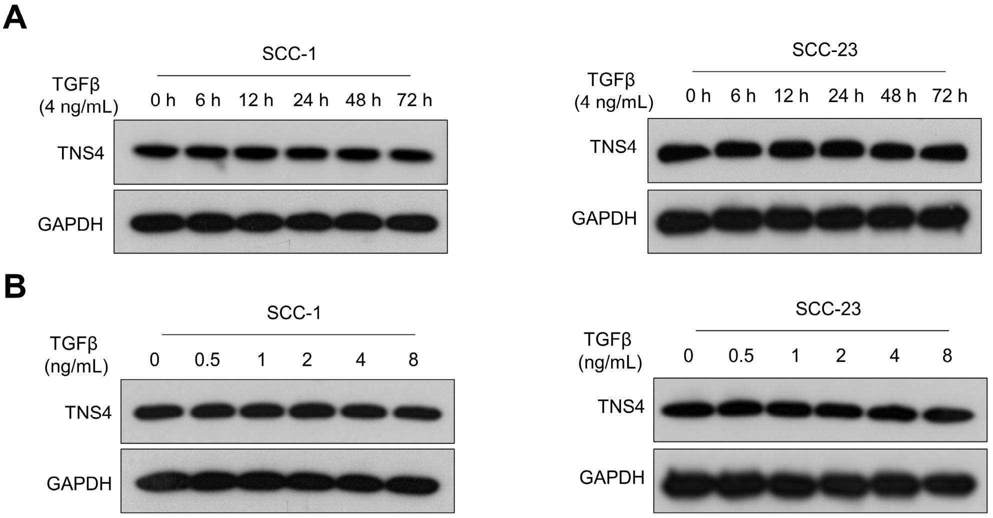

Figure S11. Impact of TGFβ supplementation on TNS4 expression level in HNSCC cells. (A) Temporal variation in TNS4 expression following different durations of TGFβ exposure. (B) Dose-dependent effects of TGFβ on TNS4 expression levels in HNSCC cells.

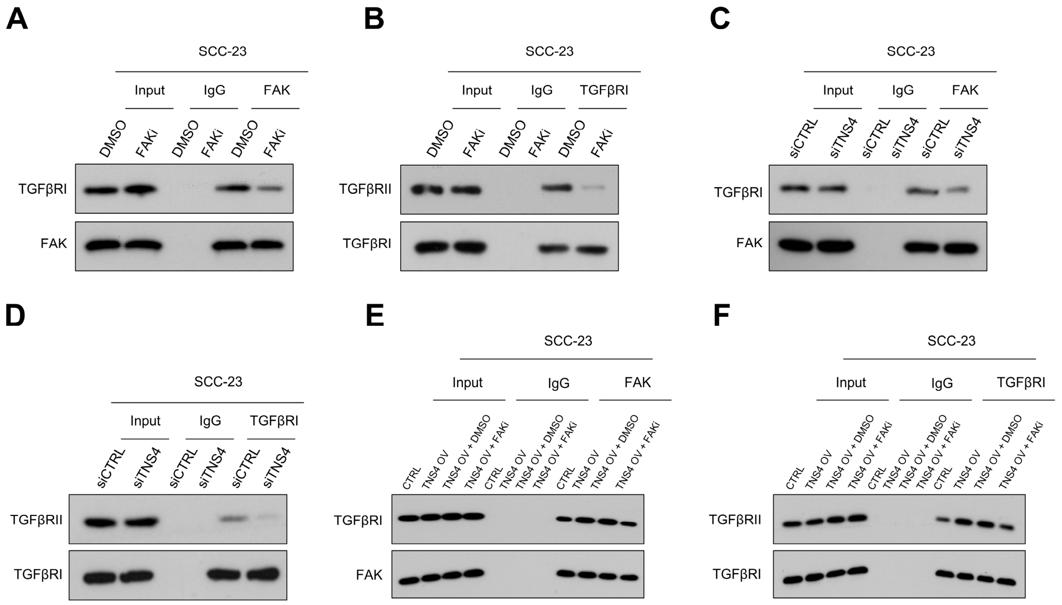

Figure S12. The TNS4-FAK axis enhances TGFβ signaling by facilitating the interaction between TGFβRI and TGFβRII in SCC-23 cells. (A-B) Analysis of the FAK inhibitor's impact on the interaction between FAK and TGFβRI, and between TGFβRI and TGFβRII. (C-D) Effects of TNS4 depletion on the interaction between FAK and TGFβRI, and between TGFβRI and TGFβRII. (E-F) Effects of TNS4 overexpression on the interaction between FAK and TGFβRI, and between TGFβRI and TGFβRII.

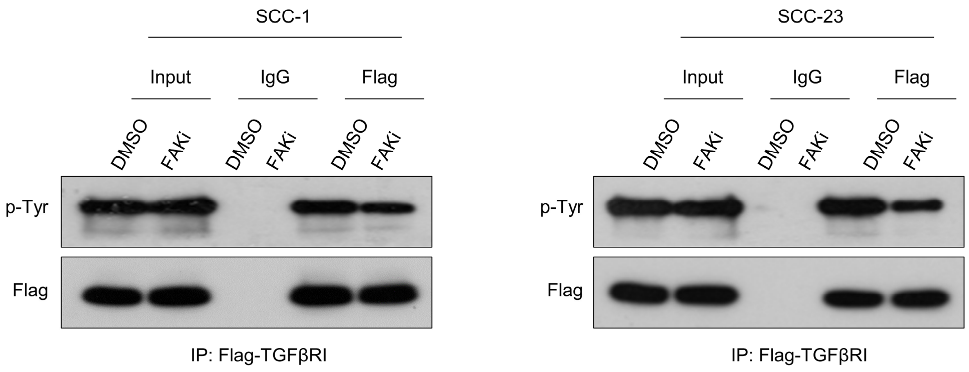

Figure S13. Inhibition of FAK markedly reduced the levels of tyrosine phosphorylation on TGFβRI in both SCC-1 and SCC-23 cell lines.

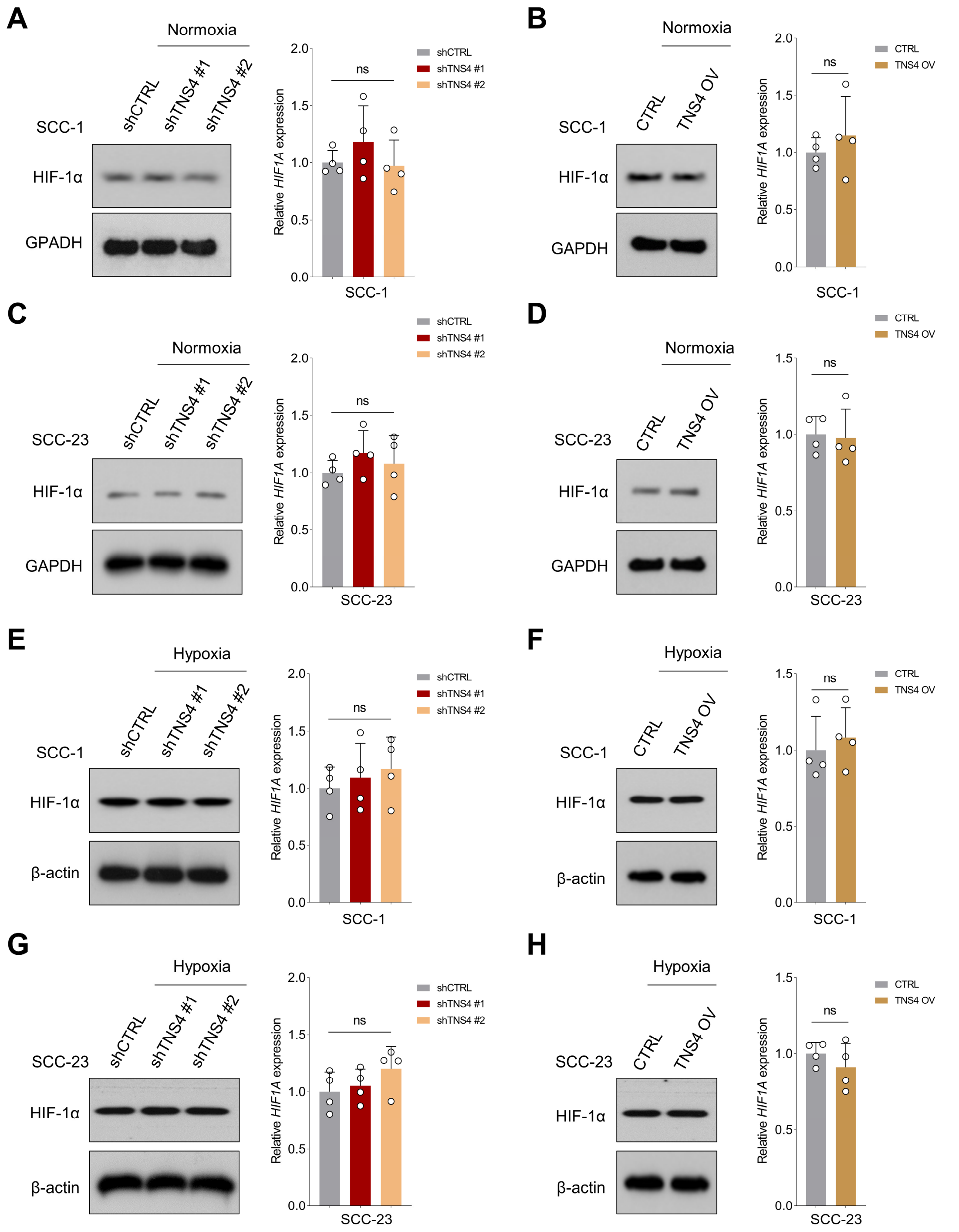

Figure S14. Effects of TNS4 depletion or overexpression on HIF-1α expression in normoxic and hypoxic conditions. (A-D) Comparative analysis of HIF-1α expression in TNS4-depleted and TNS4-overexpressing cells relative to their respective control cells under normoxic conditions. (E-H) Comparative analysis of HIF-1α expression in TNS4-depleted and TNS4-overexpressing cells relative to their respective control cells under hypoxic conditions.

Table S1. The clinicopathological information of in-house HNSCC cohort.

Clinicopathological parameters	Number of patients (n, %)
**Age**	
≥60	68 (56.20%)
<60	53 (43.80%)
**Gender**	
Male	85 (70.25%)
Female	36 (29.75%)
**Smoking**	
Yes	72 (59.50%)
No	49 (40.50%)
**Alcohol intake**	
Yes	45 (37.19%)
No	76 (62.81%)
**TNM stage**	
I-II	66 (54.55%)
III-IV	55 (45.45%)
**Lymph node metastasis**	
Negative	81 (66.94%)
Positive	40 (33.06%)

Table S2. Sequences of primers and oligos used in this study.

Gene/Oligos	Sequence (5′-3′)
TNS4	F: AGGACACCAGAACTCCGTTCA
	R: TCTCGGGTGATGTTTGGCTTA
GAPDH	F: TGCACCACCAACTGCTTAGC
	R: GGCATGGACTGTGGTCATGAG
ACTB	F: CACCATTGGCAATGAGCGGTTC
	R: AGGTCTTTGCGGATGTCCACGT
siHIF-1α	CUGAUGACCAGCAACUUGATT
shTNS4 #1	GGACCTTGACTCCTACATTGA
shTNS4 #2	GAAGTGGCAGAAGTACTGCAA
